# Evaluation of Antimicrobial, Anticholinesterase Potential of Indole Derivatives and Unexpectedly Synthesized Novel Benzodiazine: Characterization, DFT and Hirshfeld Charge Analysis

**DOI:** 10.3390/molecules28135024

**Published:** 2023-06-27

**Authors:** Abdul Rauf Raza, Syeda Laila Rubab, Muhammad Ashfaq, Yasir Altaf, Muhammad Nawaz Tahir, Muhammad Fayyaz ur Rehman, Tariq Aziz, Metab Alharbi, Abdullah F. Alasmari

**Affiliations:** 1 Institute of Chemistry, Ibn e Sina Block, University of Sargodha, Sargodha 40100, Pakistan; 2 Department of Chemistry, Division of Science and Technology, University of Education, Lahore 54770, Pakistan; 3 Department of Physics, University of Sargodha, Sargodha 40100, Pakistan; 4 School of Chemical and Physical Sciences, Victoria University of Wellington, Wellington 6140, New Zealand; 5 Department of Agriculture, University of Ioannina, 471 32 Arta, Greece; 6 Department of Pharmacology and Toxicology, College of Pharmacy, King Saud University, P.O. Box 2455, Riyadh 11451, Saudi Arabia

**Keywords:** anticholinesterase, benzodiazine, DFT, antimicrobial, indole, novel benzodiazine

## Abstract

The pharmacological effectiveness of indoles, benzoxazepines and benzodiazepines initiated our synthesis of indole fused benoxazepine/benzodiazepine heterocycles, along with enhanced biological usefulness of the fused rings. Activated indoles **5**, **6** and **7** were synthesized using modified Bischler indole synthesis rearrangement. Indole **5** was substituted with the trichloroacetyl group at the C^7^ position, yielding **8,** exclusively due to the increased nucleophilic character of C^7^. When trichloroacylated indole **8** was treated with basified ethanol or excess amminia, indole acid **9** and amide **10** were yielded, respectively. Indole amide **10** was expected to give indole fused benoxazepine/benzodiazepine **11a**/**11b** on treatment with alpha halo ester followed by a coupling agent, but when the reaction was tried, an unexpectedly rearranged novel product, 1,3-bezodiazine **12,** was obtained. The synthetic compounds were screened for anticholinesterase and antibacterial potential; results showed all products to be very important candidates for both activities, and their potential can be explored further. In addition, 1,3-bezodiazine **12** was explored by DFT studies, Hirshfeld surface charge analysis and structural insight to obrain a good picture of the structure and reactivity of the products for the design of derivatised drugs from the novel compound.

## 1. Introduction

Indoles are promising scaffolds in the medicinal world. Due to versatile applications of natural and synthetic indole derivatives in biological and pharmacological fields, they are being used as drugs, e.g., anti-HIV [1], anticancer [2,3], antimicrobial [4], antioxidant [5,6], anti-inflammatory [7], anticholinesterase [8], antidepressant [9], antihistaminic [10], anti-Parkinson [11], antitumor [12,13], tubulin inhibitors [14] and receptor inhibitors [15]. We have already successfully synthesized and published many series of indole derivatives. Like 5-membered heterocycles (indoles), 7-membered heterocycles, including benzodiazepines and benzoxazepines, are also of great medicinal importance [16]. Many benzodiazepine based drugs are being used for the treatment of alcohol withdrawal, tension, anxiety disorders [17,18], acute repetitive seizures (ARS) [19], sleep disorders and schizophrenia. We have also synthesized and published optically active benoxazepine rings successfully.

Our interest in the potent efficacy of indole reactive sites led us to explore the introduction of another ring to the indole nucleus in our current study. Specifically, we aimed to synthesize indole-based benzoxazepine/benzodiazepine compounds with enhanced pharmacological potential by incorporating both the indole and benzoxazepine/benzodiazepine cores. However, rather than obtaining the anticipated indole-based benzoxazepine/benzodiazepine compounds, we discovered a novel and interesting product, 1,3-benzodiazine, which showed promising pharmacological properties. 1,3 diazanaphthalene (quinazoline) is a significant family of heterocyclic motifs in medicine and pharmaceuticals [20]. A number of quinazoline-based drugs are known for their anti-fungal [21], anti-tubercular [22], anti-cancer [23] and anti-inflammatory properties [24].

Recently, we have reported that some indoles with anti-cholinesterase activity are comparable to standard drugs. These compounds can be explored as a good alternative in AD therapeutics [8]. All synthesized indoles and 1,3-diazine were screened for their anti-cholinesterase potential. There is a continuous need to develop new antibacterial drugs to address the growing problem of emerging pathogenic infections and antibiotic resistance. Given the ability of indoles to combat bacterial infections, various types of indole derivatives have been explored for their potential antibacterial activities. Based on indoles’ capability to fight against bacteria, natural indole alkaloids [25], bis-indoles [26], indole-containing hybrid [27], spirocyclic [28], and many other indole derivatives are being used as potential antibacterial agents. Regarding the significant antibacterial potential of indoles, synthesized rings are tested for antibacterial potential.

Considering this significance, the current study aims to conduct Hirshfeld surface analysis and Density Functional Theory (DFT) studies of the novel indole-fused benzodiazine compound (**12**). By conducting in-depth analyses (of specific characteristics and chemical reactivity) of this novel compound (indole-fused benzodiazine), we can better understand its potential as an effective pharmaceutical candidate and ultimately contribute to developing novel treatments for various diseases.

## 2. Results and Discussions

### 2.1. Synthesis

**Scheme 1:**
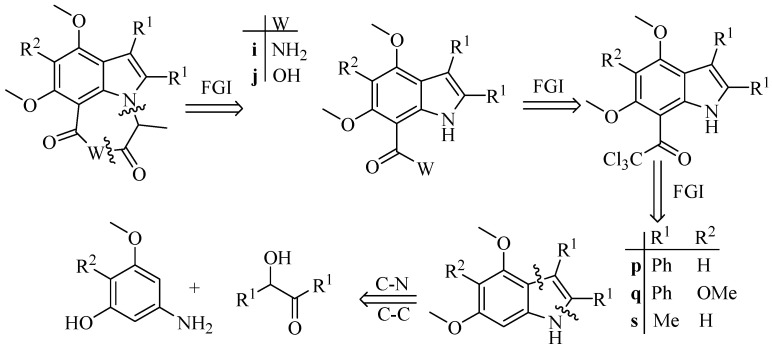
Retrosynthesis of indole-based benzodiazepine/benzoxazepine.

Owing to its pharmacological potential, it was planned to synthesize indole-based benzodiazepine/benzoxazepine. The retrosynthesis for the target compound is given in Scheme 1.

**Scheme 2:**
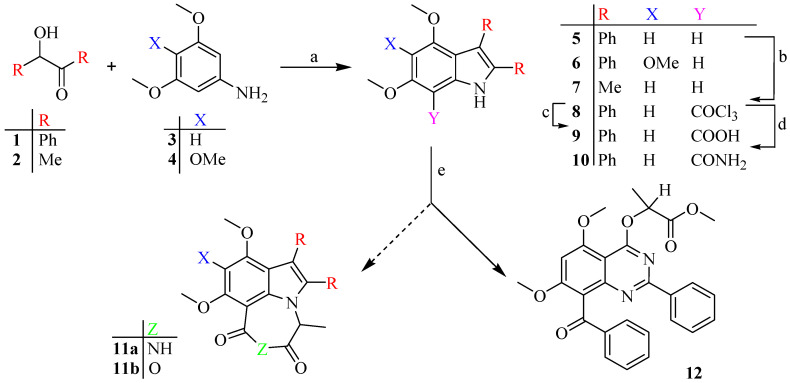
Reagents and conditions: (**a**) PhNH_2_, AcOH or NaHCO_3_ (LiBr) reflux in EtOH; (**b**) Cl_3_CCOCl_,_ CH_2_Cl_2_, reflux; (**c**) aq KOH in EtOH; (**d**) aq NH_3_, reflux; (**e**) NaH in DMSO (solid arrow), MeCH(Br)CO_2_Me (arrow with dashed lines).

We synthesized indoles (**5**–**7**) following our already-reported procedure [29]. The indoles (**5**–**7**) (Figures S1–S3) are activated and make only the C^7^ highly electron-rich position, which acts as a good nucleophile and can undergo aromatic substitution reactions. The indole **5** was reacted with Cl_3_CCOCl in CH_2_Cl_2_ to afford trichloroacetyl substituted indole **8** (Figure S4). When indole **8** in CH_2_Cl_2_ was stirred with *aq*. KOH or liquid NH_3_, this resulted in indole acid **9** (Figure S5) and indole amide **10** (Figure S6), respectively [30]. We attempted to convert the indole amide **10** into indole-fused benzodiazepine-1,3-dione (**11a**–**b**) by reacting with a coupling agent methyl 2-bromomethyl propionate (Scheme 2).

**Figure 1:**
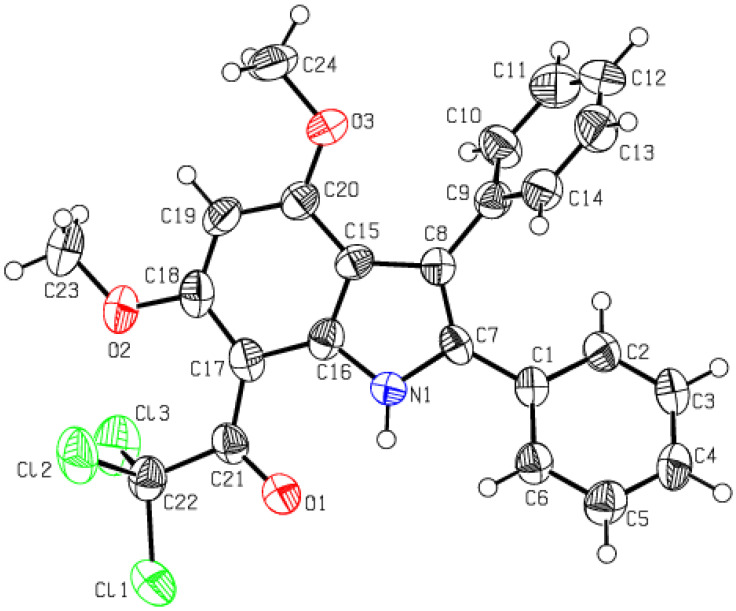
The ORTEP presentation of **8** formed at a probability level of 50%. Hollow circles represent H-atoms (black balls shows carbon atoms, red balls show oxygen, while blue and green show nitrogen and chloride, respectively).

When **5** was refluxed with Cl_3_CCOCl in CH_2_Cl_2_, the proton with indolic C^7^ of **5** was substituted by a trichloro-acetyl group. The ^1^H NMR of **8** showed the disappearance of H^7^; the C^7^ shifted downfield compared to the reactant, and a new carbonyl CO (C^8^) signal emerged in the ^13^C NMR spectrum of **8**. These changes indicated the conversion of **5** to **8**. Further confirmation was achieved by ESI MS of the **8** exhibiting molecular ion [M + H]^+•^ at 474.0432 amu (found for C_24_H_19_^35^Cl_3_NO_3_) and the XRD structure was also proof of COCCl_3_ substitution (Figure 1) (File S1).

**Scheme 3:**
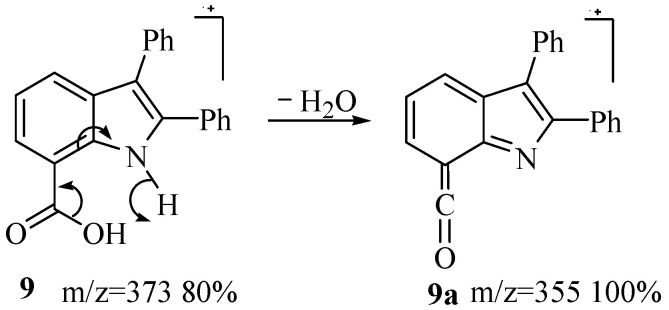
LR EIMS fragmentation of **9**.

The trichlor-acetyl group, being a good leaving group, was conveniently substituted by OH or NH_3_ to produce indole acid **9** or amide **10,** accordingly, by refluxing indole **8** with basified EtOH or excess aqueous NH_3_ in THF. The amide formation was authenticated by the appearance of two amide protons in the ^1^H NMR. One proton emerged as a doublet (*J* = 3.0 Hz) at 7.81 ppm, and the other proton appeared as a singlet at 7.54 ppm. The D_2_O exchange experiments also verified the formation of carboxylic acid **9**/unsubstituted acid amide **10**. Additionally, the ^13^C NMR showed a C^8^ signal of C=O in **9** and **10,** shifted upfield to 166.9 and 169.5 ppm, respectively, from the ketonic C^8^ signal in **8** at 182.0 ppm. This was clear evidence for the substitution of the (COCCl_3_) group, having a strong –*I* effect, by a group with a greater +*R* effect. A single (M)^+•^ peak in the LR EIMS of **9** at 373 amu also assured the stability of the target molecule and the conversion of **8** to **9**. The fragment **9a** was produced after H_2_O loss and β–ketoacid-type rearrangement, which afforded a base peak at 355 amu (Scheme 3). The ultimate proof was provided by CHNS analysis, which showed comparable percentages for all elements.

**Figure 2:**
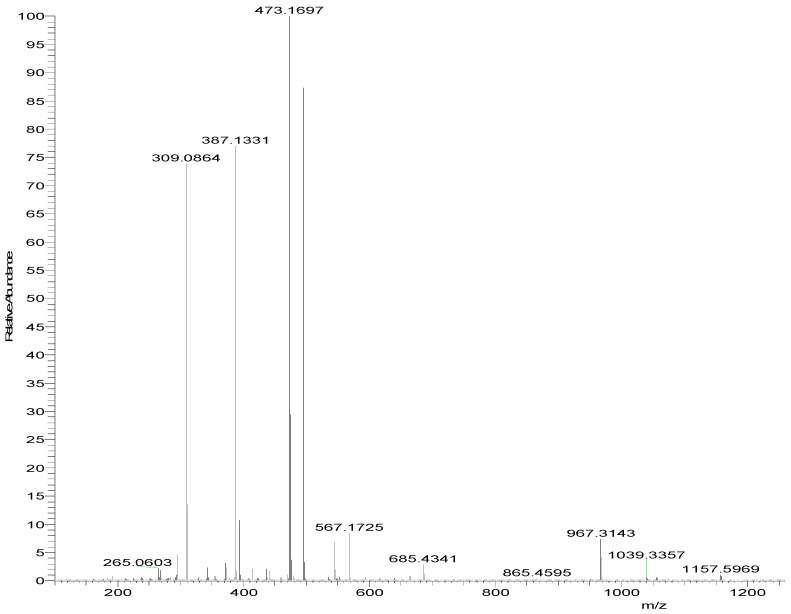
A portion of ESI MS of **12** showing (M + H)^+•^.

The indole amide **10** was coupled with ester, methyl 2-bromopropanoate by deprotonating amidic and indolic NH of **10** with the subsequent addition of a coupling reagent to yield **11a**. A strong base NaH in anhydrous DMSO was used for deprotonation, followed by refluxing of the reaction mixture at 170 °C for 2 h. The reaction was cooled before the addition of propanoate, followed by reflux. A mixture of products was obtained, rendered to column chromatography, and a white powder **12** was separated. The ^1^H NMR of **12** (Figure S7) showed all the expected aliphatic peaks and indole ring peaks, indicating the attachment of **10** with ester. It appeared that we had successfully synthesized **11a**, but the ^13^C NMR of **12** (Figure S8) showed a few unjustified signals. The ESI MS of the product showed (M + H)^+•^ at 473.1697 amu, which did not agree **11a** with the expected (M)^+•^ (426.46 amu for **11a**) (Figure 2).

**Figure 3:**
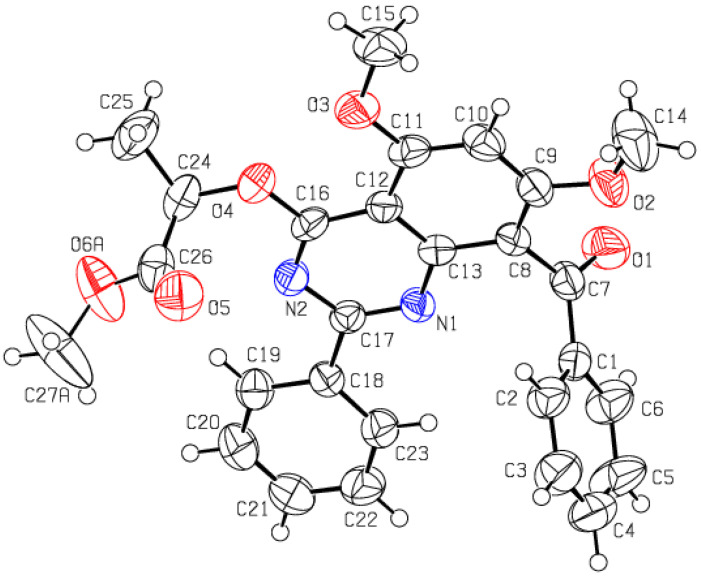
The ORTEP presentation of **12** formed at a probability level of 50%. Hollow circles represent H-atoms (black balls shows carbon atoms, red balls show oxygen, while blue show nitrogen).

However, the single crystal XRD (Figure S9, File S2) of the product unveiled the mystery, showing that **12** was a novel rearranged indole based 1,3-diazine (Figure 3).

### 2.2. DFT Studies

**Figure 4:**
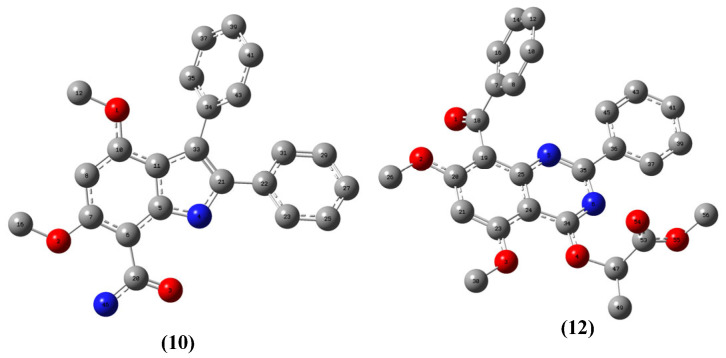
Optimized geometries of compounds (**10**) and (**12**) at PBE0-D3BJ/def2-TZVP level of theory. In 3D models, red represents oxygen, grey carbon, and blue nitrogen atoms. Hydrogen atoms are omitted for clarity. (Grey balls shows carbon atoms, red balls show oxygen, while show nitrogen).

Figure 4 shows the optimized geometries of (**10**) and (**12**) generated through GaussView. These structures are true minima, as shown by the absence of any imaginary frequency. Comparing the calculated structure with the crystallographic information shows that both results correlate well. For instance, in (**12**), C25-N3 is 1.365 (3) Å while C34-N6 is 1.310 (4) Å in the crystal structure. The computational bond lengths of these two bonds are 1.358 Å and 1.307 Å, respectively. Therefore, negligible deviations in computational results from the crystal structure can be seen. Such a comparison is not possible for compound (**10**) since we do not have its crystal structure.

#### Frontier Molecular Orbital (FMO) Analysis and Hyperpolarizability

**Figure 5:**
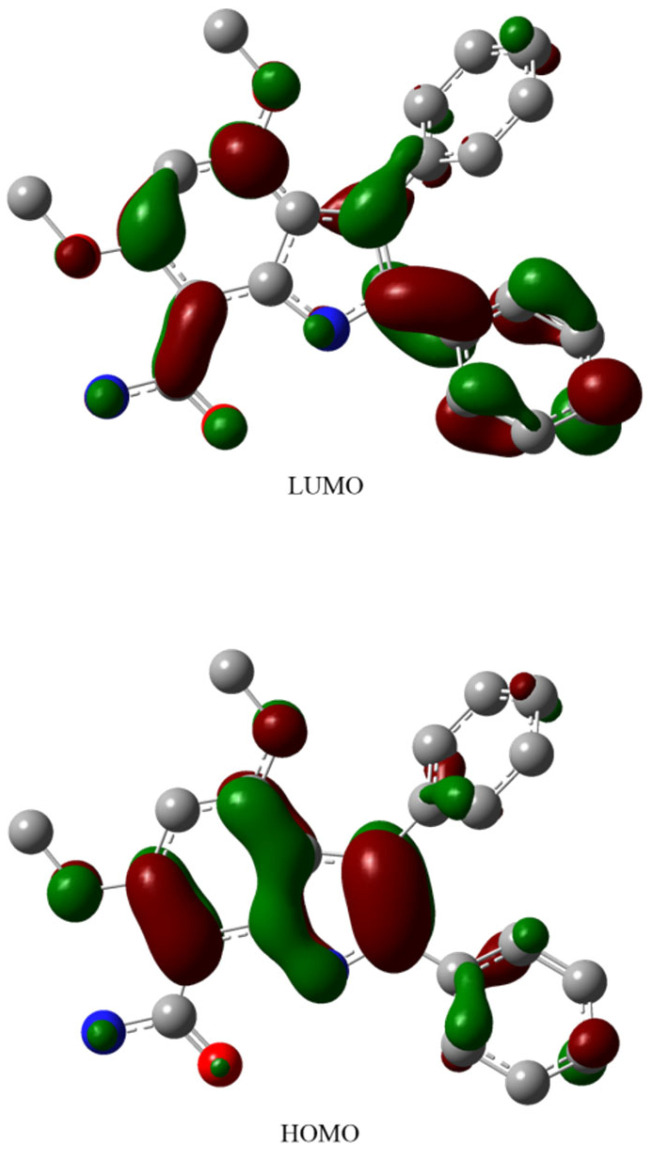
Iso-surface lots of frontier molecular orbitals of compound (**10**).

Reactivity and other physical parameters, such as the energies of the highest occupied molecular orbital (HOMO) and the lowest unoccupied molecular orbitals (LUMO), can be analyzed through frontier molecular orbital analysis [31]. In our case, since compound (**10**) exhibited an unexpected reactivity, its FMO analysis will be helpful in obtaining a better insight. Its calculated HOMO-LUMO energy difference is 4.51 eV. Figure 5 shows the iso-surface plots of HOMO and LUMO of compound (**10**). We can see that HOMO is present on the main indole framework, LUMO is on the right-side aromatic moiety, and the amido group is on the indole. Both compounds (**10**) and (**12**) are expected to show excellent non-linear optical properties, as indicated by their hyperpolarizability (*β*) of 4643.02 a.u. and 1009.56 a.u., respectively.

### 2.3. Crystal Data

#### 2.3.1. Single Crystal XRD Depiction of **8** and **12**

**Figure 6:**
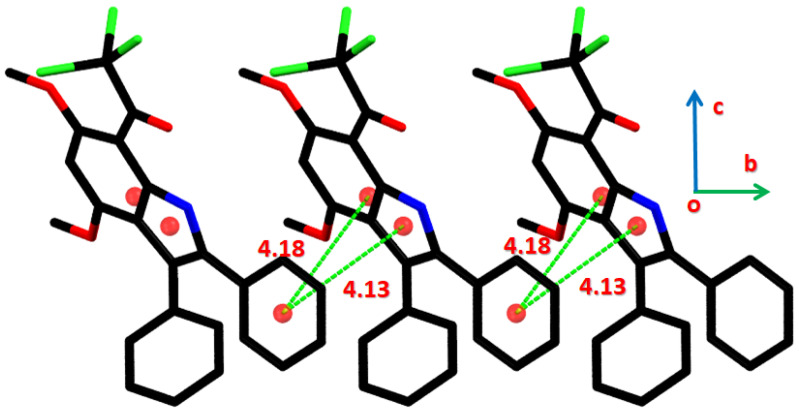
Graphical illustration of π⋯π stacking interaction in the supramolecular assembly of **8**. For clarity, H-atoms are not displayed.

In the crystal structure of (Figure 1 and Table 1), the 4,6-dimethoxy-1H-indole-7-carbaldehyde group A (C7/C8/C15-C24/N1/O1-O3) is planar with root mean square deviation of 0.0426 Å. The phenyl rings B (C1–C6) and C (C9–C14) attached to group A are inclined at the dihedral angles of 7.39 (5)° and 59.16 (8)°. The bond lengths and angles of significant importance are consistent with the corresponding ones in the literature compounds [32,33]. The configuration of the molecule is stabilized by intra-molecular N-H⋯O bonding. Hydrogen bonding is not present in the supramolecular assembly. The molecules are connected through π⋯π stacking interaction along *b*-axis. The phenyl ring (C1–C6) of a molecule interacts with the 2,3-dihydro-1H-pyrrole and 1H-indole rings of the neighboring symmetry-related molecule with separation between the centers of interacting ring range from 4.13 to 4.18 Å (Figure 6). The dihedral angles between the interacting rings range from 29.6 (4)° to 31.4 (3)°. The supramolecular assembly is further stabilized by weak C-H⋯π and C-Cl⋯π interactions with an H⋯π distance of 2.68 Å, whereas Cl⋯π has a distance of 5.718 (8) Å.

In the crystal structure of (Figure 3 and Table 1), the quinazoline ring A (C8–C13/C16/C17/N1/N2) is planar with a root mean square deviation of 0.0207 Å. The methoxy group B (C14/O2) has a larger deviation from the plane of group A compared to the other methoxy group C (C15/O3). The benzaldehyde group D (C1–C7/O1) and phenyl ring E (C18–C23) are inclined at the dihedral angles of 9.96 (4)° and 10.59 (1)° with respect to group A. The methoxy group of methyl 2-hydroxypropanoate moiety F (C24–C26/C27A/O4/O5/O6A) is disordered over two sites and is stabilized by using various suitable restraints.

**Figure 7:**
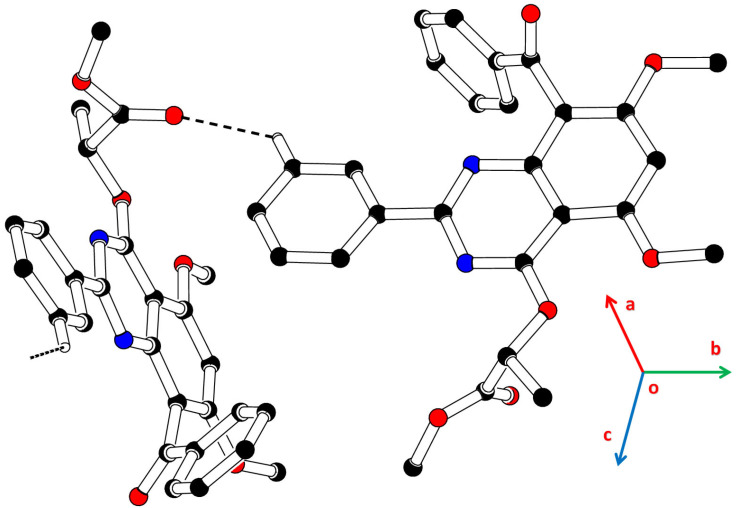
Packing diagram of **12** showing infinite chain formed by H-bonding. For clarity, selected H-atoms are displayed.

**Figure 8:**
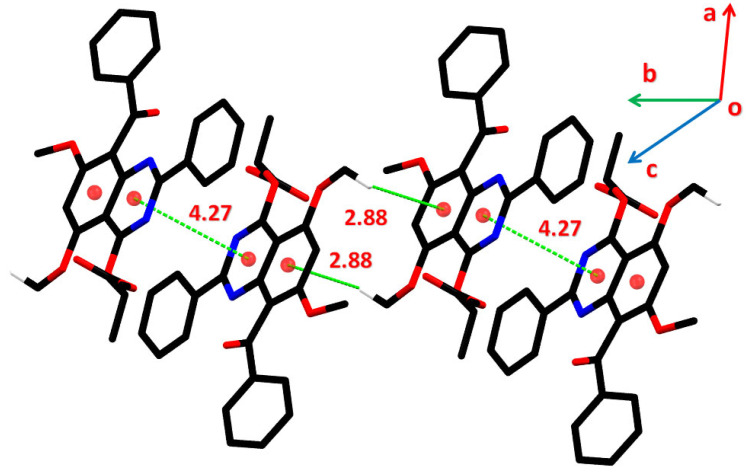
Graphical illustration of π⋯π stacking and C-H⋯π interaction in the supramolecular assembly of **12**. For clarity, H-atoms are not displayed.

The bond lengths and angles of significant importance are consistent with the corresponding ones in the literature compounds [34,35,36]. The molecules are connected in the form of a C(11) chain by C-H⋯O bonding, where CH is from ring E, and carbonyl O-atom of moiety F acts as an H-bond acceptor (Figure 7). The chain runs propagate along the b-axis. The supramolecular assembly is further stabilized by offset π⋯π stacking interactions among the pyrimidine rings of the symmetry-related molecules and C-H⋯π interaction with CH from the methoxy group C (Figure 8). The dihedral angle between the symmetry-related pyrimidine rings is 0.03 (13)° with an inter-centroid separation of 4.27 Å.

#### 2.3.2. Hirshfeld Surface Analysis

**Figure 9:**
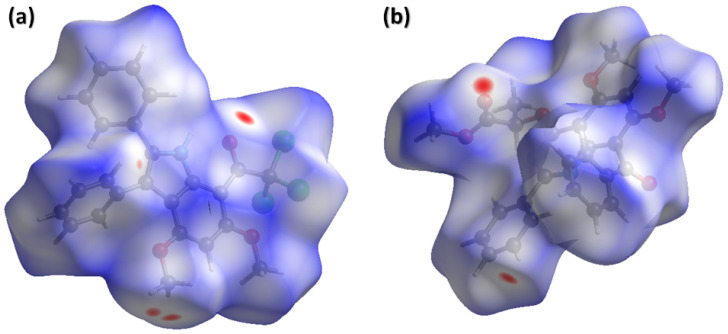
Hirshfeld surface plotted over for (**a**) **8** in the range −0.1019 to 1.4971 a.u, (**b**) **12** in the range −0.1593 to 1.8443 a.u.

Supramolecular chemistry is deeply focused on understanding the intermolecular interactions in the crystals, because these interactions are the decider of the properties. In this scenario, Hirshfeld surface analysis is performed using Crystal Explorer version 21.5 [37] to inspect the intermolecular interactions. The short contacts and comparatively longer contacts present in the crystal can be recognized by plotting Hirshfeld surface over $d_{norm}$. The surface consists of red, blue and white colors. Red and blue regions show short and long contacts, respectively. The contacts with a distance equal to the sum of the van der Waal radii are shown by white regions [38]. Figure 9a,b shows the Hirshfeld surface over $d_{norm}$ for **8** and **12**, respectively. For **8**, the red spot on the surface around carbonyl O-atom showed short O⋯Cl contact and red spots around CH of the methoxy group showed short H⋯C contacts (Figure 9a). For **12**, the red spot on the surface around carbonyl O-atom showed short O⋯H contact and red spots around CH of the phenyl ring showed short H⋯O contact (Figure 9b).

**Figure 10:**
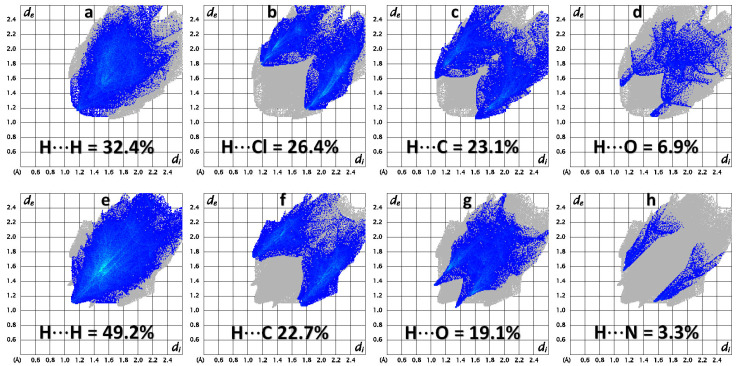
Important 2D plots for (**a**–**d**) **8**, (**e**–**h**) **12** (Figure S10).

**Figure 11:**
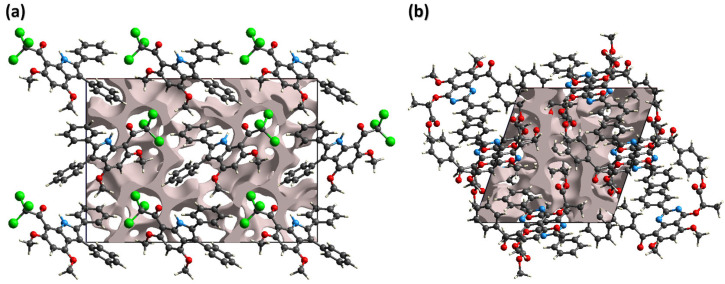
Graphical view of voids along the *b*-axis for (**a**) LR-III92A, (**b**) LR-III-9730_0m.

The contribution of the contact to the stabilization of the supramolecular assembly of the crystal can be determined and displayed by 2D fingerprint plots [39]. Figure 10a–h show the 2D plots of significant importance for **8** and **12**, respectively. For both compounds, H⋯H contacts make the largest contribution to the supramolecular assembly, with a percentage contribution of 32.4% in **8** (Figure 10a) and 49.2% in **12** (Figure 10e). The next important contact for **8** is H⋯Cl, with a contribution of 26.4% (Figure 11b), whereas the crystal structure of **12** contained no chlorine atoms, so there are no contacts involving the chlorine atom. O⋯H contact makes a larger contribution to **12** as compared to **8,** as in the absence of chlorine atoms, oxygen atoms contribute more to the stabilization of supramolecular assembly. H⋯C contact makes a significant contribution to both compounds, as both have a number of short H⋯C contacts.

The crystal’s response to applied stress depends on the voids present in it. A crystal with large cavities cannot bear a significant amount of stress. In order to check the mechanical strength of **8** and **12**, we calculate voids using an approach reported in the literature [40]. The pro-crystal electron density idea is used to calculate voids with an iso-surface of 0.0002 a.u (Figure 11). The volume of voids is 334.69 and 399.09 Å^3^ in **8** and **12**, respectively. The voids consume 15.1% of the space in **8** and 16.4% in **12**. The voids occupied a small space in both compounds, so there is no large cavity, and compounds are expected to have a good mechanical response.

### 2.4. In Silico Anticholinesterase Activity

**Figure 12:**
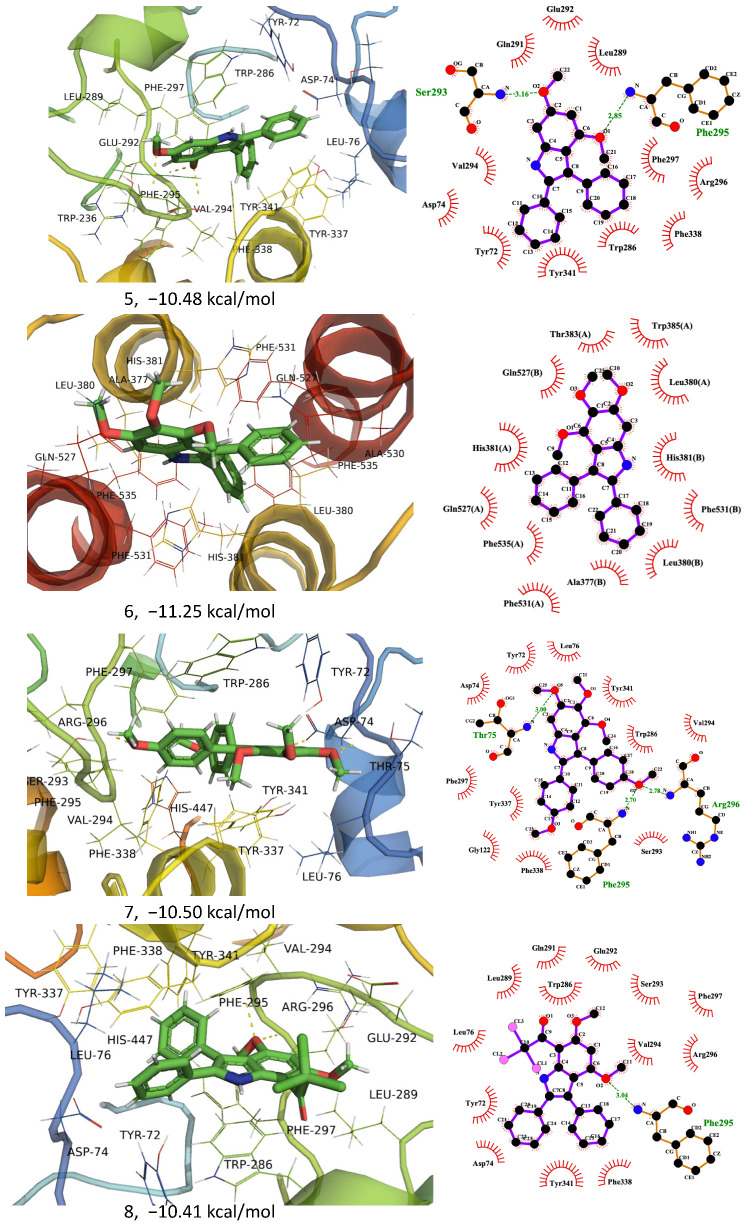

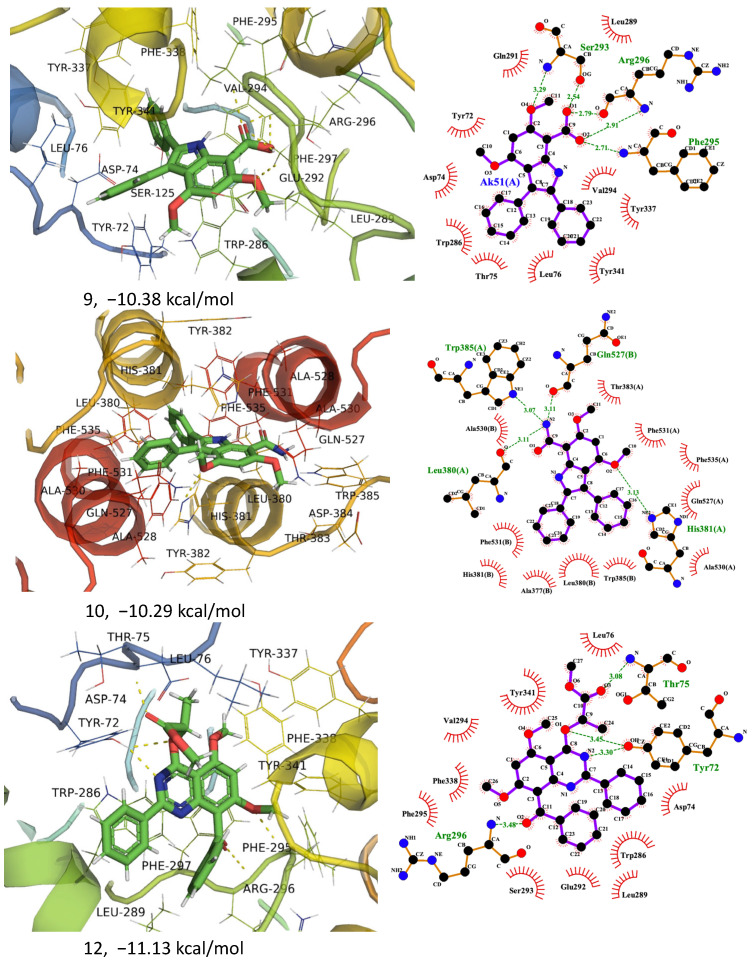
Ligand protein interactions for the proposed compounds (**5**–**10**, **12**). The right panels show the ligand interactions in the binding pocket (prepared using PyMol), while the left panels show H bonding and hydrophobic interactions.

Virtual screening of the proposed compounds (**5**–**10** and **12**) was performed against Human Acetylcholinesterase (hAChE). All compounds show very good binding to hAChE. A dimeric form of the enzyme was used for the docking study, and it was very interesting to note that 6 and 10 showed binding to the dimeric interface of the enzyme (ALA377 (Chain A), LE 380 (Chain A), HIS381 (Chain A), THR383 (Chain A), ASP384 (Chain A), TRP385, (Chain A), GLN527 (Chain A), ALA530 (Chain A), PHE531 (Chain A), PHE535 (Chain B), ALA377 (Chain B), VAL378 (Chain B), LEU380 (Chain B), HIS381 (Chain B), THR383 (Chain B), TRP385 (Chain B), GLN527 (Chain B), ALA530 (Chain B). PHE531 (Chain B) and PHE535) with binding energies of −11.25 and −10.46, respectively (Figure 12 and Table 2).

All other ligands preferably bind to the peripheral anionic site (PAS) of acetylcholinesterase, consisting of TYR72, ASP74, TYR124, TRP286, and TYR341 amino acids. The PAS site is connected to the active site residue of the enzyme SER203, GLU334, and HIS447 by a narrow path. Ligand 7 shows the best binding to PAS residue, including TYR72, ASP74, THR75, LE 76, THR83, TRP286, HIS287, LEU289, GLN291, GLU292, SER293, VAL294, PHE295, ARG296, PHE297, TYR337, PHE338, TYR341, GLY342 (Figure 12) with binding energy and dissociation constant of −11.13 kcal/mol and 6.97 nM, respectively (Table 2). Four H-bonds were observed, i.e., two with TYR72 (3.3 and 3.45 Å), one with each THR75 (3.08 Å) and ARG296 (3.48 Å) (Figure 12). A π–π stacking was also observed with TRP286, which has been reported to impair enzyme activity. The PHE297 from the acyl pocket of the enzyme is also surrounded by the ligand and may involve in impairing its interactions with its substrate acetyl group. Previously, TRP286 and VAL294 were found to interact with substituted benzene rings in azine derivatives by π-π- and π-alkyl hydrophobic interactions [41].

Comparable binding energies were calculated for other ligands (5, 7–9, 12) to the same PAS binding site, where active site residues include TYR72, THR75, ASP74, TRP286, LEU76, LEU289, GLU292, GLN291, SER293, ARG296, VAL294, PHE297, PHE295, TYR337, TYR341 and PHE338, amino acids. Ligand 8 shows a binding energy value of −10.46 kcal/mol and a constant dissociation value of 21.69 nM (Table 2). It stabilizes its binding with three hydrogen bonds, each with THR75, PHE295, and ARG296 (Figure 12). In vitro and in vivo studies are required for further exploration and confirmation of compounds and anticholinesterase inhibitors, as in silico activities only suggest/predict the expected biological potential of the synthetic compounds.

### 2.5. Anticholinesterase and Antibacterial Activities

Azine derivatives, benzoxazepines and benzodiazepines show various biological activities, including antibacterial, antifungal, and anticholinesterase activities [42,43,44]. This makes benzo-diazine an excellent candidate for exploration of its potential for cholinesterase inhibition and antimicrobial activities. The synthesized compounds (**8**, **9**, **10** and **12**) were subjected to anti-acetylcholinesterase activity, and **8** and **12** showed better acetylcholinesterase inhibition in comparison to other compounds, showing IC_50_ values of 7.31 and 6.11 μM, respectively, but due to solubility issues the IC_50_ values can be far less than the apparent value. The same can be true for **9** and **12**, precipitated even at lower concentrations in the reaction mixture. The standard drug galantamine IC_50_ value was 5.32 μM. The in silico results also agree with these findings and show very low dissociation constants and strong binding energies. In addition, 4,6-Dimethoxyindole-based Azines derivative showed a maximum of 30–64% inhibition of acetylcholinesterase while using 200 µM compounds, where dimethoxy substituted compounds were shown to be the best inhibitor [41]. Some azine Schiff bases showed an IC_50_ of 23.60 ± 0.63 µg/mL and 28.59 ± 0.07 µg/mL [45].

Disc diffusion assay was performed to evaluate the antibacterial potential of compounds (**8**, **9**, **10** and **12**) against antibiotic-resistant strains of *Salmonella Typhi* and *Staphylococcus aureus.* Only **12** showed considerable antibacterial activity against *S. Typhi* (ZOI ~13 mm), while against *S. aureus,* all four compounds (**8**, **9**, **10** and **12**) showed very good antibacterial activity showing zone of inhibition (ZOI) as ~18, 21, 17 and 20 mm, respectively. Heterocyclic compounds are well-known for their antibacterial potential. A series of Benzoxazepine derivatives have shown promising antibacterial activities against *Staphylococcus sciuri* (zone of inhibition as 8–21 mm) and *E. coli* (zone of inhibition as 8–14 mm) [46]. Isoxazolyl benzo [1, 4]diazepine-5-ones have shown antibacterial against both G + ve (*B. subtilis*, *B. sphaericus*, *S.aureus*) and G-ve bacteria (*P. aeruginosa*, *K. aerogenes*, *C. violaceum*) [47]. Benzoxazepine, benzothiazepine and benzodiazepine derivatives showed good antibacterial activity. The zone of inhibition was found to be 1–15 mm for *E. coli*, 12–18 mm for *B. subtilis*, 10–20 for *S. aureus* and 10–22 for *P. aeruginosa* [48].

## 3. Materials and Methods

### 3.1. Synthesis

#### 3.1.1. General Procedure for the Synthesis Indoles, **5**–**6**

A mixture of benzoin (2-hydroxy-1,2-diphenylethanone) and 3,4,5-trimethoxy/3,5-dimethoxyaniline (3 eq, 13.1 mmol) was heated for about 2.5 h at 120 °C. The mixture was allowed to cool at room temperature, followed by the addition of Acetic acid (AcOH) (32 eq, 0.141 mol, 8.5 g, 8.1 mL) and aniline (1 eq, 4.4 mmol, 0.41 mL). After mixing, the resulting combination was heated for 5 h at 130 °C. The mixture was again allowed to cool at room temperature and then filtered. In order to obtain a white solid (50–60%), the crude product was washed with methanol (MeOH)

#### 3.1.2. 4,6-Dimethoxy-2,3-Diphenyl-1H-Indole, **5**

3,5-Dimethoxyaniline (3 eq, 13.1 mmol, 2.01 g); 2-hydroxy-1,2-diphenylethanone (3 eq, 13.1 mmol, 2.8 g) colorless solid (56%, 2.4 g); Rf is 0.3 (*n*-hexane/EtOAc 3:2); Melting Point: 240–242 °C; UV (λ*_max_*, nm, log ε)) (325, 4.62403), (274, 5.56330); IR (ύ_max_, cm^−1^) KBr: (N–H) 3343; ^1^H NMR (δ in ppm, CDCl_3_, 300 MHz): 8.16 (1H, NH, bs), 745–722 (10 H, 2 ph, m), 6.57 (1H, H^7^, *J* = 1.9 Hz, m) 6.26 (1H, H^5^, *J* = 1.9 Hz, d) 3.92, 3.73 (3H each, OCH_3_, s); ^13^C NMR (δ in ppm, CDCl_3_, 75 MHz): 157.8, 155.3 (C^6^ & C^4^, both s), 137.4, 135.9 (C^7a^ & C^2^, both s), 133.0, 131.9 (C^1^′ & C^1^″, both s), 131.5, 128.5, 127.8, 127.3 (all 2×, C^3^″, C^2^″, C^3^′ & C^2^′, all d), 126.9, 125.9 (C^4^″, C^4^′, both d), 115.0, 113.0 (C^3a^ & C^3^, both s) 92.5 86.5 (C^7^ & C^5^, both d), 55.7, 55.2 (OCH_3_, q); LR EIMS (amu, *m*/*z*) 314 (54%) [M − Me^•^]^+^, 329 (100%) [M]^+•^; CHNS analysis: (C_22_H_19_NO_2_): 79.9% C, 5.7% H, 4.2% N, requires: 80.2% C, 5.8% H, 4.3% N.

#### 3.1.3. 4,5,6-Trimethoxy-2,3-Diphenyl-1H-Indole, **6**

2-hydroxy-1,2-diphenylethanone (3 eq, 13.1 mmol, 2.8 g), 3,4,5-Trimethoxyaniline (3 eq, 13.1 mmol, 2.40 g); crystalline solid in off white colour (50%, 2.4 g); R*_f_* is 0.25 (*n*-hexane/EtOAc, 7:1); Melting Point: 218–220 °C; UV: (λ*_max_* in nm, log ε): (318, 2.89228); IR: (ύ_max_ in cm^−1^) KBr: 3363 for N–H; ^1^H NMR: [(δ (ppm, CD_3_)_2_SO, 300 MHz)]: 8.12 (1H, NH, bs), 7.48–7.23 (10 H, 2 Ph, both m), 6.78 (1H, H^7^, s) 3.87, 3.72. 3.36 (3H each, OCH_3_, all s), ^13^C NMR [(CD_3_)_2_SO, 75MHz, δ (ppm)]: 151.0, 146.7, 136.8 (C^6^, C^5^ & C^4^, all s), 136.6, 133.4 (C^7a^ & C^2^, both s), 133.0, 132.9 (C^1^″ & C^1^′, both s), 131.3, 128.0, 128.7, 127.9 (all 2×, C^3^″, C^3^′, C^2^″ & C^2^′, all d) 127.2, 126.4 (C^4^″ & C^4^′, both d), 115.8, 113.5 (C^3a^ & C^3^, both s), 90.7 (C^7^, s) 61.2, 60.9, 56.3 (OCH_3_, q); LR EIMS (amu, *m*/*z*): 359 (100%) [M]^+•^.

#### 3.1.4. 4,6-Dimethoxy-2,3-Dimethyl -1H-Indole, **7**

3-chlorobutanone (10.0 mmol, 1.06 g, 1eq), 3,5-dimethoxyaniline (10 mmol, 1.53 g, 1 eq); NaHCO_3_ (19.0 mmol, 1.6 g, 1.9 eq) and LiBr (13 mmol, 1.1 g, 1.3 eq) in absolute EtOH (20 mL) were refluxed for 3 h. After cooling to ambient, the resulting solid was separated by filtration and washed with H_2_O to give 7, a white solid (50% yield). M.P.: 115–116 °C [31]; IR: (ύ_max_ in cm^−1^) KBr: 3382 for N–H; ^1^H NMR [δ in ppm, 300 MHz, (CHCl_3_)]: 7.565 (1H, *indolic* NH, s), 6.39 (1H, H^7^, s), 6.18 (1H, H^5^, s), 3.89–3.84 (3H each, OCH_3_, s), 2.36, 2.29 (3H each, CH_3_, s).

#### 3.1.5. 7-Trichloro-acetyl-4,6-Dimethoxy-2,3-Diphenyl-1H-Indole, **8**

A mixture of Trichloro-acetyl chloride (Cl_3_CCOCl) (1.5 eq, 4.95 mmol, 0.90 g, 0.56 mL) and 5 indole (1 eq, 3.3 mmol, 1.08 g) was refluxed for 3 h in dichloromethane (CH_2_Cl_2_) (25 mL). Ice cold water (80 mL) was used to quench the reaction. The mixture was extracted with dichloromethane (CH_2_Cl_2_) (3 × 15 mL). 354, a yellow solid (65%, 1.01 g) was obtained by drying the combined organic layers over anhydrous Na_2_SO_4,_ followed by in vacuo concentration.

R*_f_* is 0.57 (*n*-hexane/EtOAc, 3:1); Melting Point: 176–177 ° [32]; UV: (λ*_max_* in nm, log ε): (310, 4.27256), (270, 4.3460); IR: (ύ_max_ in cm^−1^) KBr: (C–Cl) 693, (N–H) 3052, (C=O) 1632; ^1^H NMR: [δ(ppm) CDCl_3_, 300MHz]: 10.44 (1H, NH, bs), 7.39–7.22 (10 H, 2 Ph, m), 6.19 (1H, H^5^, s), 3.99, 3.82 (3H each, OCH_3_, both s); ^13^C NMR: [δ(ppm), CDCl_3_, 75 MHz]: 182.0 (C^8^, s), 161.7, 160.2 (C^6^ & C^4^, both s), 138.9, 135.2 (C^7a^ & C^2^, both s), 132.9, 131.9 (C^1^′ & C^1^″, both s), 131.2, 128.4, 127.8, 127.4 (all 2×, C^3^″,C^2^″, C^3^′ & C^2^′, all d), 127.3, 126.3 (C^4^″ & C^4^′, both d), 115.2, 113.4 (C^3a^ & C^3^, both s), 98.6, 98.2 (C^7^ & C^9^, both s), 87.7 (C^5^, d), 55.5, 55.4 (OCH_3_, q); ESI MS: amu (C_24_H_19_^35^Cl_3_NO_3_), [M + H]^+•^ observed (474.0432), calculated (474.0431), variation (0.1 mmu).

#### 3.1.6. 4,6-Dimethoxy-2,3-Diphenylindole-7-Carboxylic Acid, **9**

7-trichloroacetylindole 8 (1 eq, 5.3 mmol 2.52 g) was refluxed in the presence of KOH (7.1 eq, 37.63 mmol, 2.1 g) in the *aq.* EtOH (1:1, 50 mL) for 10 min. Then Cooled and neutralized with HCl (10%) to have the acid 355 (80%, 1.59 g). R*_f_* is 0.2 (*n*-hexane/EtOAc, 3:1); Melting Point: 190–192 °C; IR (cm^−1^, ύ_max_) KBr: (C=O) 1627, (bs,O–H, N–H) 3340; ^1^H NMR (δ in ppm, 300 MHz, CDCl_3_): bs 10.9 (1H, COOH), 10.6 (1H, NH, bs), 7.40–7.10 (10H, 2 Ph, m), 6.30 (1H, H^5^, s), 4.15, 3.80 (3H each, OCH_3_, s), ^13^C NMR (δ in ppm, 75 MHz, CDCl_3_): 166.9 (C^8^, s), 159.4, 157.5 (C^6^ & C^4^, both s), 138.1,135.4 (C^7a^ & C^2^, both s), 134.0, 132.1 (C^1^′ & C^1^″, both s), 131.4, 128.5, 128.1, 127.5 (all 2×, C^3^″, C^2^′, C^3^′ & C^2^, all d), 127.3, 126.3 (C^4^″ & C^4^, both d), 114.4, 114.2 (C^3a^ & C^3^, both s), 94.1 (C^7^, s), 87.1 (C^5^, d), 57.5, 55.5 (OCH_3_,q),; LR EIMS (amu, *m*/*z*): [M]^+•^ 373 (80%), [M − H_2_O]^+•^ 355 (100%); CHNS analysis: for C_23_H_19_NO_4_: 73.6% C; 4.8% H, 3.5% N, requires; 74.0% C, 5.1% H & 3.7% N.

#### 3.1.7. 4,6-Dimethoxy-2,3-Diphenyl-1H-Indole-7-Carboxamide, **10**

A solution of 7-trichloroacetylindole 8 (2.7 mmol, 1.28 g) in 10 mL THF was allowed to react with an excess of *aq.* NH_3_ (1:4, 20 mL) for 30 min. After the reaction completion, the solvent was rotary evaporated, then chilled with ice-cold water, and the resultant mixture was filtered to obtain the amide 41 (80%, 0.80 g). R*_f_* is 0.4 (*n*-hexane/EtOAc, 3:1); MP is 176 °C; UV: log ε (nm, λ*_max_*): 3.76554 (337), 4.53951 (274); IR (cm^−1^, ύ_max_) KBr: 3832, 3732 (NH_2_), 3176 (N–H), 1575 (C=O, amide); ^1^H NMR [δ in ppm, 300 MHz, (CD_3_)_2_SO]: 11.36 (1H, indolic NH, s), 7.81 (1H, *J* = 3 Hz, amidic NH, d), 7.54 (1H, *amidic* NH, s), 7.24–7.31 (10H, 2 Ph, m), 6.47 (1H, H^5^, s), 4.05, 3.76 (3H each, OCH_3_, both s); ^13^C NMR [75 MHz, δ in ppm, (CD_3_)_2_O,]: 169.5 (C^8^, s), 158.1, 157.2 (C^6^ & C^4^, both s), 138.7, 135.9 (C^7a^ & C^2^, both s), 133.4,132.6 (C^1^′, C^1^″, both s), 131.5, 128.4, 128.1, 127.4 (all 2×, C^3^″, C^2^″, C^3^′ & C^2^′, all d), 127.1, 126.0 (C^4^″ & C^4^′, both d), 113.8, 113.7 (C^3a^ & C^3^, both s), 96.8 (C^7^, s), 87.6 (C^5^, s) 56.9, 55.3 (OCH_3_, both q).

#### 3.1.8. 8-Benzoyl-5,7-Dimethoxy-4-(1‴-Methyoxycarbonylethoxy)-2-Phenyl-1,3-Benzodiazine, **12**

Indole amide 10 (1 eq, 0.8 mmol, 0.30 g) was added to the mixture of 2ml DMSO and NaH (2 eq, 1.6 mmol, 40 mg) with continuous stirring for 1.5 h at temp. 160 °C, then cooled, methyl bromo-propanoate (1.2 eq, 0.96 mmol, 0.11 mL, 0.16 g) was added and stirred overnight. Then, 100 mL H_2_O was added, the resultant solid was first filtered, dried and then flashed chromatographed with 5, 10, 15, 20, 25, 30% × 7 (100 mL each) EtOAc in *n*-hexane, to obtain a white solid (39%, 0.15 g) in 63rd to 96th fraction, each of 25 mL. R*_f_* is 0.4 (1:1, *n*-hexane/EtOAc); Melting Point: 120 °C; UV: (log ε nm, λ_max_): (4.27047, 278); IR (cm^−1^, ύ_max_) KBr: 1755 (ester, C=O), 1681 (ketone, C=O), 1577 (C=N); ^1^H NMR (δ in ppm, 300 MHz, CDCl_3_): 8.11–7.88 (5H, Bz, m), 7.55–7.30 (5H, Ph, m), 6.66 (1H, H^6^, s), 5.52 (IH, OCH, *J* = 7.2 Hz, q), 4.10 (3H, COOCH_3_, s), 3.95, 3.76 (3H each, OCH_3_, both s), 1.81 (3H, CH_3_, *J* = 7.2 Hz, d); ^13^C NMR (δ in ppm, 75 MHz, CDCl_3_,): 196.0 (C^9^, s), 171.2 (C^2^, s), 172.6 (C^1‴^, s), 161.2, 160.1 (C^7^ & C^5^, both s), 159.4, (C^8a^, s), 153.3 (C^4^, s), 138.9, 137.1 (C^1^′, C^1^″, both s), 129.4 (2×, C^2^″, C^4^″, d), 128.4 (2×, C^3^′, d), 128.3 (2×, C^3^′, d), 128.0 (2×, C^2^′, C^4^′, d), 116.2, 100.9 (C^8^ & C^4a^, both s), 93.7 (C^6^, d), 71.5 (C^1^‴, d), 56.2, 56.5 (OCH_3_, both q), 52.1 (CO_2_CH_3_, q), 17.3 (CH_3_, q); ESI MS [M + H]^+•^ (amu): 473.1712 (calc.), 473.1697 (found), 1.5 mmu difference.

### 3.2. Computational Methods

Computational insight has been helpful in studying geometric features and reactivity of molecules for a long time. Here, we used computational methods employing density functional tools, as implemented in the Gaussian 09 [49] suite of programs. We applied the PBE0 hybrid functional method [50,51] along with the def2-TZVP basis set of triple ζ quality [52] in our calculations. Grimme’s empirical dispersion correction with Becke-Johnston damping (D3BJ) [53,54,55] was added to count for dispersion interactions. The optimized geometries were confirmed to be the true minima, as indicated by the frequency output where no imaginary frequency was found. Molecular docking was used to probe ligand–protein interaction against Human Acetylcholinesterase (PDB ID 4MOE), as described previously by our group. YASARA Dynamics was employed with the parameters used before [56,57]. The visualisation and figures were prepared using PyMol [58,59,60] and LigPlot [61].

### 3.3. Antibacterial and Anticholinesterase Activities

Antibacterial activity was assessed against clinically isolated, antibiotic-resistant *strains of Salmonella typhi* and *Staphylococcus aureus* using Disk diffusion assay, as previously described [62]. Anticholinesterase activity was also performed, as described earlier for indoles.

### 3.4. Single Crystal XRD Details of ***10*** and ***12***

The crystals of suitable size are selected and mounted on Bruker Kappa Apex-II for the sake of data collection, as molybdenum X-ray source. For the structure solution and refinement, SHELXT-2014 [61] and SHELXL-2019/2 [63] software were used. Non-hydrogen atoms and H-atoms were assigned anisotropic displacement (parameters and isotropic displacement parameters, respectively). For eye-catching graphics, PLATON [64] and Mercury [65,66] software were employed.

## 4. Conclusions

During trials to synthesize pharmacologically potent indole fused benoxazepine/benzodiazepine heterocycles, an unexpectedly rearranged novel product, 1,3-bezodiazine, was obtained. Compounds (**10**) and (**12**) were subject to computational insight (DFT studies and Hirshfeld surface charge analysis) for a better understanding of geometric features and reactivity of the products, to create an idea for the design of derivative drugs from the novel compound. FMO analysis revealed that LUMO is present over amido function, which is expected to undergo internal rearrangement. An excellent nonlinear optical response is expected as a result of significant hyperpolarizability. The synthesized compounds, particularly 3-bezodiazine, have shown considerable anticholinesterase and antibacterial activities, and these can be explored further for in vitro and in vivo activities and toxicity at the cellular level.

## Figures and Tables

**Table 1:** Experimentally related important details for **8** and **12**.

CCDC		
Chemical formula	C_24_H_18_Cl_3_NO_3_	C_27_H_24_N_2_O_6_
*M*_r_	474.74	472.48
Crystal system, space group	Orthorhombic, *Pca*2_1_	Monoclinic, P2_1_/n
a, b, c (Å)	22.879 (5), 6.0315 (14), 16.084 (4)	14.3363 (16), 12.0884 (13), 14.8394 (17)
Temperature (K)	296	296
V (Å^3^)	2219.5 (9)	2430.8 (5)
α, β, γ (°)	90, 90, 90	90, 109.053 (6), 90
Z	4	4
µ (mm^−1^)	0.44	0.09
Radiation type	Mo *K*α	Mo *K*α
Crystal size (mm)	0.36 × 0.18 × 0.15	0.35 × 0.30 × 0.24
**Data Collection**		
Absorption correction	Multi-scan (SADABS; Bruker, 2007)	Multi-scan (*SADABS*; Bruker, 2007)
No. of observed, independent and measured [*I* > 2σ (*I*)] reflections	1588, 3218, 9325	1588, 3218, 9325
(sin θ/λ)_max_ (Å^−1^)	0.606	0.617
Diffractometer	Bruker Kappa APEXII CCD	Bruker Kappa APEXII CCD
R_int_	0.095	0.083
**Refinement**		
No. of reflections	3218	4764
No. of restraints	1	26
No. of parameters	270	341
*R* [*F*^2^ > 2σ(*F*^2^)], w*R*(*F*^2^), *S*	0.045, 0.091, 0.97	0.058, 0.144, 0.99
Δρ_max_, Δρ_min_ (e Å^−3^)	0.20, −0.24	0.16, −0.16
H-atom treatment	H-atom parameters constrained	H-atom parameters constrained

**Table 2:** Binding energies, dissociation constants and binding site residues for the ligands (**5**–**10**, **12**).

Compounds	Binding Energy (kcal/mol)	Dissociation Constants (nM)	Binding Sites
**5**	−10.48	20.74	TYR 72, THR 75, ASP 74, LEU 76, LEU 289, TRP 286, GLN 291, SER 293, GLU 292, VAL 294, ARG 296, PHE 295, PHE 297, PHE 338, TYR 337, TYR 341
**6**	−11.25	5.67	ALA 377 (Chain A), LEU 380 (Chain A), HIS 381 (Chain A), THR 383 (Chain A), ASP 384 (Chain A), TRP 385, (Chain A), GLN 527 (Chain A), ALA 530 (Chain A), PHE 531 (Chain A), PHE 535 (Chain B), ALA 377 (Chain B), VAL 378 (Chain B), LEU 380 (Chain B), HIS 381 (Chain B), THR 383 (Chain B), TRP 385 (Chain B), GLN 527 (Chain B), ALA 530 (Chain B). PHE 531 (Chain B), PHE 535
	−9.79	66.97	TYR 72, THR 75, ASP 74, LEU 76, GLY 121, SER 125, GLY 122, TRP 286, PHE 295, ARG 296, VAL 294, TYR 337, PHE 297, TYR 341, PHE 338, HIS 447
**7**	−10.50	20.13	TYR 72, ASP 74, THR 75, LEU 76, GLY 121, GLY 122, SER 125, SER 203, TRP 286, LEU 289, SER 293, VAL 294, PHE 295, ARG 296, PHE 297, TYR 337, PHE 338, TYR 341, HIS 447
**8**	−10.46	21.69	TYR 72, THR 75, ASP 74, LEU 76, LEU 289, TRP 286, GLN 291, SER 293, GLU 292, VAL 294, ARG 296, PHE 295, PHE 297, PHE 338, TYR 337, TYR 341
**9**	−10.38	24.58	TYR 72, TYR 341, ASP 74, LEU 76, THR 75, TRP 286, GLN 291, LEU 289, SER 293, GLU 292, PHE 295, VAL 294, ARG 296, TYR 337, PHE 297, PHE 338,
**10**	−10.46	21.49	ALA 377 (Chain A), LEU 380 (Chain A), HIS 381 (Chain A), THR 383 (Chain A), ASP 384 (Chain A), TRP 385, (Chain A), GLN 527 (Chain A), ALA 530 (Chain A), PHE 531 (Chain A), PHE 535 (Chain B), ALA 377 (Chain B), VAL 378 (Chain B), LEU 380 (Chain B), HIS 381 (Chain B), THR 383 (Chain B), TRP 385 (Chain B), GLN 527 (Chain B), ALA 530 (Chain B). PHE 531 (Chain B), PHE 535
	−10.29	28.76	TYR 341, PHE 338, TYR 337, PHE 297, ARG 296, PHE 295, VAL 294, SER 293, GLU 292, GLN 291, LEU 289, LEU 76, ASP 74, THR 75, TYR 72
**12**	−11.13	6.97	TYR 72, ASP 74, THR 75, TRP 286, THR 83, LEU 76, HIS 287, TRP 286, LEU 289, GLU 292, GLN 291, VAL 294, PHE 295, SER 293, ARG 296, TYR 337, PHE 297, PHE 338, GLY 342, TYR 341

## Data Availability

The samples of the compound and docking datasets may be available from corresponding authors on request.

## Supplementary Materials

The following supporting information can be downloaded at: https://www.mdpi.com/article/10.3390/molecules28135024/s1, Figure S1: ^1^HNMR Structure **5**, Figure S2: ^1^HNMR Structure **7**, Figure S3: ^13^CNMR Structure **7**, Figure S4: ^1^HNMR Structure **8**, Figure S5: ^1^HNMR Structure **9**, Figure S6: ^13^CNMR Structure **10**, Figure S7: ^1^HNMR Structure **12**, Figure S8: ^13^CNMR Structure **12**, Figure S9: MS Data from Orbitrap Structure **12**, Figure S10: Remaining 2 D plots of (**a**–**d**) **8**, (**e**–**h**) **12**, File S1: checkCIF/PLATON report structure **8**, File S2: checkCIF/PLATON report structure 12.

## Sample Availability

Sample are available on request.

## References

[1] Chen Q., Wu C., Zhu J., Li E., Xu Z. (2022). Therapeutic potential of indole derivatives as anti-HIV agents: A mini-review. Curr. Top. Med. Chem..

[2] Sachdeva H., Mathur J., Guleria A. (2020). Indole derivatives as potential anticancer agents: A review. J. Chil. Chem. Soc..

[3] Devi N., Kaur K., Biharee A., Jaitak V. (2021). Recent development in indole derivatives as anticancer agent: A mechanistic approach. Anti-Cancer Agents Med. Chem..

[4] Sayed M., Kamal El-Dean A.M., Ahmed M., Hassanien R. (2019). Design, synthesis, and characterization of novel pyrimidines bearing indole as antimicrobial agents. J. Chin. Chem. Soc..

[5] Kherkhache H., Benabdelaziz I., Silva A.M., Lahrech M.B., Benalia M., Haba H. (2020). A new indole alkaloid, antioxidant and antibacterial activities of crude extracts from *Saccocalyx satureioides*. Nat. Prod. Res..

[6] Rubab S.L., Nisar B., Raza A.R., Saadia M., Tahir M.N., Sajjad N., Shajahan S., Sharmila V., Acevedo R. (2022). Synthesis and antioxidant screening of Novel indole amines. J. Iran. Chem. Soc..

[7] Singh S., Sharma N., Chandra R. (2022). The indole nucleus as a selective COX-2 inhibitor and anti-inflammatory agent (2011–2022). Org. Chem. Front..

[8] Gondal H.Y., Tariq S., Akhter S., Raza A.R., Ur Rehman M.F., Rubab S.L.J.R.A. (2023). Synthesis, characterization, and in vitro anti-cholinesterase screening of novel indole amines. RSC Adv..

[9] Kumar R.R., Kumar V., Kaur D., Nandi N.K., Dwivedi A.R., Kumar V., Kumar B.J.C. (2021). Investigation of indole-3-piperazinyl derivatives as potential antidepressants: Design, synthesis, in-vitro, in-vivo and in-silico analysis. ChemistrySelect.

[10] Sravanthi T., Manju S. (2016). Indoles—A promising scaffold for drug development. Eur. J. Pharm. Sci..

[11] Borg H.M., Kabel A., Abdel-Kareem M. (2020). Effect of metformin and indole-3-carbinol on a rat model of Parkinson’s disease induced by 6-hydroxydopamine. Bull. Egypt. Soc. Physiol. Sci..

[12] Han Y., Dong W., Guo Q., Li X., Huang L. (2020). The importance of indole and azaindole scaffold in the development of antitumor agents. Eur. J. Med. Chem..

[13] Chen F.-Y., Li X., Zhu H.-P., Huang W. (2020). Regulation of the ras-related signaling pathway by small molecules containing an indole core scaffold: A potential antitumor therapy. Front. Pharmacol..

[14] Tang S., Zhou Z., Jiang Z., Zhu W., Qiao D.J.M. (2022). Indole-Based Tubulin Inhibitors: Binding Modes and SARs Investigations. Molecules.

[15] Mohamed F.A.M., Alakilli S.Y.M., El Azab E.F., Baawad F.A.M., Shaaban E.I.A., Alrub H.A., Hendawy O., Gomaa H.A.M., Bakr A.G., Abdelrahman M.H. (2023). Discovery of new 5-substituted-indole-2-carboxamides as dual epidermal growth factor receptor (EGFR)/cyclin dependent kinase-2 (CDK2) inhibitors with potent antiproliferative action. RSC Med. Chem..

[16] Bhathiwal A.S., Bendi A., Tiwari A. (2022). A study on synthesis of benzodiazepine scaffolds using biologically active chalcones as precursors. J. Mol. Struct..

[17] Ahwazi H.H., Abdijadid S. (2019). Chlordiazepoxide. https://europepmc.org/article/NBK/nbk547659.

[18] Singh R., Abdijadid S. (2019). Oxazepam. https://europepmc.org/article/NBK/nbk544349.

[19] Boddu S.H., Kumari S.J.P. (2020). A short review on the intranasal delivery of diazepam for treating acute repetitive seizures. Pharmaceutics.

[20] Mathew T., Papp A.Á., Paknia F., Fustero S., Prakash G.S. (2017). Benzodiazines: Recent synthetic advances. Chem. Soc. Rev..

[21] Liu F., Huang Y. (2011). Physiology. Antifungal bioactivity of 6-bromo-4-ethoxyethylthio quinazoline. Pestic. Biochem. Physiol..

[22] Kuneš J., Bažant J., Pour M., Waisser K., Šlosárek M., Janota J. (2000). Quinazoline derivatives with antitubercular activity. Il Farm..

[23] Marzaro G., Guiotto A., Chilin A. (2012). Quinazoline derivatives as potential anticancer agents: A patent review (2007–2010). Expert Opin. Ther. Patents.

[24] Alafeefy A.M., Kadi A.A., Al-Deeb O.A., El-Tahir K.E., Al-Jaber N.A. (2010). Synthesis, analgesic and anti-inflammatory evaluation of some novel quinazoline derivatives. Eur. J. Med. Chem..

[25] Yan Y., Li X., Zhang C., Lv L., Gao B., Li M. (2021). Research progress on antibacterial activities and mechanisms of natural alkaloids: A review. Antibiotics.

[26] Qin H.-L., Liu J., Fang W.-Y., Ravindar L., Rakesh K. (2020). Indole-based derivatives as potential antibacterial activity against methicillin-resistance Staphylococcus aureus (MRSA). Eur. J. Med. Chem..

[27] Song F., Li Z., Bian Y., Huo X., Fang J., Shao L., Zhou M. (2020). Indole/isatin-containing hybrids as potential antibacterial agents. Arch. Pharm..

[28] Ciulla M.G., Kumar K. (2018). The natural and synthetic indole weaponry against bacteria. Tetrahedron Lett..

[29] Phuntsho N. (2014). The Electrochemistry and Fluorescence Studies of 2, 3-Diphenyl Indole Derivatives. Ph.D. Thesis.

[30] Wood K., Black D.S., Kumar N.J.T. (2011). Ring closing metathesis strategies towards functionalised 1, 7-annulated 4, 6-dimethoxyindoles. Tetrahedron.

[31] Arshad M.N., Bibi A., Mahmood T., Asiri A.M., Ayub K. (2015). Synthesis, crystal structures and spectroscopic properties of triazine-based hydrazone derivatives; a comparative experimental-theoretical study. Molecules.

[32] Beyer R.L., Kandemir H., Bhadbhade M., Sengul I.F., Leu C.-w., Wenholz D., Kumar N., Black D.S. (2018). Synthesis of dipyrrolo [2, 3-a: 1′, 2′, 3′-fg] acridin-12 (1H)-ones. Tetrahedron Lett..

[33] Corey E., Link J.O., Sarshar S., Shao Y. (1992). X-ray diffraction studies of crystalline trihalomethyl ketones (RCOCX3) reveal an unusual structural deformation about the carbonyl group. Tetrahedron Lett..

[34] Wang X., Lerchen A., Glorius F. (2016). A comparative investigation: Group 9 Cp* M (III)-catalyzed formal [4 + 2] cycloaddition as an atom-economic approach to quinazolines. Org. Lett..

[35] Wang J., Zha S., Chen K., Zhang F., Song C., Zhu J. (2016). Quinazoline synthesis via Rh (III)-catalyzed intermolecular C–H functionalization of benzimidates with dioxazolones. Org. Lett..

[36] Lingayya R., Vellakkaran M., Nagaiah K., Tadikamalla P.R., Nanubolu J.B. (2017). Palladium (II)-catalyzed direct O-alkenylation of 2-arylquinazolinones with N-tosylhydrazones: An efficient route to O-alkenylquinazolines. Chem. Commun..

[37] Spackman P.R., Turner M.J., McKinnon J.J., Wolff S.K., Grimwood D.J., Jayatilaka D., Spackman M. (2021). A32. CrystalExplorer: A program for Hirshfeld surface analysis, visualization and quantitative analysis of molecular crystals. J. Appl. Crystallogr..

[38] Spackman M.A., Jayatilaka D.J.C. (2009). Hirshfeld surface analysis. CrystEngComm.

[39] McKinnon J.J., Jayatilaka D., Spackman M.A. (2007). Towards quantitative analysis of intermolecular interactions with Hirshfeld surfaces. Chem. Commun..

[40] Turner M.J., McKinnon J.J., Jayatilaka D., Spackman M.A. (2011). Visualisation and characterisation of voids in crystalline materials. CrystEngComm.

[41] Bingul M., Ercan S., Boga M., Bingul A.A. (2023). Antioxidant and Anticholinesterase Potentials of Novel 4,6-Dimethoxyindole based Unsymmetrical Azines: Synthesis, Molecular Modeling, In Silico ADME Prediction and Biological Evaluations. Polycycl. Aromat. Compd..

[42] Botros S., Naguib B., Osman A. (2011). Synthesis and Tranquillizing Effect of New 1, 4-Benzoxazepines. Egypt. J. Chem..

[43] Odame F., Neglo D., Sedohia D., Arthur R. (2022). Antifungal synergistic effects and anti-biofilm formation activities of some bioactive 2,3-dihydro-1,5-benzoxazepine derivatives. Arch. Microbiol..

[44] Stefaniak M., Olszewska B. (2021). 1,5-Benzoxazepines as a unique and potent scaffold for activity drugs: A review. Archiv. Der Pharm..

[45] Chouiter A.D., Mousser M.O., Mousser H.B., Krid A., Belkhiri L., Fleutot S., François M. (2023). Synthesis, spectra, crystal, DFT, molecular docking and in vitro cholinesterase inhibition evaluation on two novel symmetrical Azine Schiff bases. J. Mol. Struct..

[46] Ayyash A.N. (2019). Design, Synthesis, and Antimicrobial Studies of Novel Derivatives: Benzoxazepine-4, 7-dione and Benzodiazepine-4, 7-dione. Indian J. Heterocycl. Chem..

[47] Ponduri R., Kumar P., Vadali L.R. (2018). Synthesis and Antimicrobial Activity of New Isoxazolyl Benzo[1, 4] diazepine-5-one Derivatives. Chem. Sel..

[48] Kendre B.V., Landge M.G., Bhusare S.R. (2019). Synthesis and biological evaluation of some novel pyrazole, isoxazole, benzoxazepine, benzothiazepine and benzodiazepine derivatives bearing an aryl sulfonate moiety as antimicrobial and anti-inflammatory agents. Arab. J. Chem..

[49] Frisch M.J., Trucks G.W., Schlegel H.B., Scuseria G.E., Robb M.A., Cheeseman J.R., Scalmani G., Barone V., Mennucci B., Petersson G.A. (2010). Gaussian 09.

[50] Perdew J.P., Burke K., Ernzerhof M. (1996). Generalized Gradient Approximation Made Simple. Phys. Rev. Lett..

[51] Perdew J.P. (1991). Generalized gradient approximations for exchange and correlation: A look backward and forward. Phys. B Condens. Matter.

[52] Weigend F., Ahlrichs R. (2005). Balanced basis sets of split valence, triple zeta valence and quadruple zeta valence quality for H to Rn: Design and assessment of accuracy. Phys. Chem. Chem. Phys..

[53] Grimme S. (2006). Semiempirical GGA-type density functional constructed with a long-range dispersion correction. J. Comput. Chem..

[54] Grimme S., Antony J., Ehrlich S., Krieg H. (2010). A consistent and accurate ab initio parametrization of density functional dispersion correction (DFT-D) for the 94 elements H-Pu. J. Chem. Phys..

[55] Grimme S., Ehrlich S., Goerigk L. (2011). Effect of the damping function in dispersion corrected density functional theory. J. Comput. Chem..

[56] Rehman M.F.U., Akhter S., Batool A.I., Selamoglu Z., Sevindik M., Eman R., Mustaqeem M., Akram M.S., Kanwal F., Lu C. (2021). Effectiveness of natural antioxidants against SARS-CoV-2? Insights from the In-Silico World. Antibiotics.

[57] Bilal S., Sami A.J., Hayat A., ur Rehman M.F. (2022). Assessment of pesticide induced inhibition of Apis mellifera (honeybee) acetylcholinesterase by means of N-doped carbon dots/BSA nanocomposite modified electrochemical biosensor. Bioelectrochemistry.

[58] DeLano W.L. (2002). Pymol: An open-source molecular graphics tool. CCP4 Newsl. Protein Crystallogr.

[59] Naveed M., Ain N.u., Aziz T., Javed K., Shabbir M.A., Alharbi M., Alsahammari A., Alasmari A.F. (2023). Artificial Intelligence Assisted Pharmacophore Design for Philadelphia Chromosome-Positive Leukemia with Gamma-Tocotrienol: A Toxicity Comparison Approach with Asciminib. Biomedicines.

[60] Naveed M., Shabbir M.A., Ain N.-u., Javed K., Mahmood S., Aziz T., Khan A.A., Nabi G., Shahzad M., Alharbi M.E. (2023). Chain-Engineering-Based De Novo Drug Design against MPXVgp169 Virulent Protein of Monkeypox Virus: A Molecular Modification Approach. Bioengineering.

[61] Wallace A.C., Laskowski R.A., Thornton J.M. (1995). LIGPLOT: A program to generate schematic diagrams of protein-ligand interactions. Protein Eng. Des. Sel..

[62] Aslam M., Shahid M., Ur Rehman F., Murtaza M.A., Sharif S., Ata A., Noor S. (2012). Production optimization and characterization of a low molecular weight bacteriocin from *Lactococcus lactis* subsp. *lactis*. Afr. J. Microbiol. Res.

[63] Sheldrick G.M. (2015). SHELXT–Integrated space-group and crystal-structure determination. Acta Crystallogr. Sect. A Found. Adv..

[64] Sheldrick G.M. (2015). Crystal structure refinement with SHELXL. Acta Crystallogr. Sect. C Struct. Chem..

[65] Spek A.L. (2009). Structure validation in chemical crystallography. Acta Crystallogr. Sect. D Biol. Crystallogr..

[66] Macrae C.F., Sovago I., Cottrell S.J., Galek P.T., McCabe P., Pidcock E., Platings M., Shields G.P., Stevens J.S., Towler M.J. (2020). Mercury 4.0: From visualization to analysis, design and prediction. J. Appl. Crystallogr..

