# Supplementary material for: Evaluation of Antimicrobial, Anticholinesterase Potential of Indole Derivatives and Unexpectedly Synthesized Novel Benzodiazine: Characterization, DFT and Hirshfeld Charge Analysis
Source: Molecules. 2023 Jun 27;28(13):5024. doi: 10.3390/molecules28135024 (PMC10343724; doi:10.3390/molecules28135024)

Supplementary Information File

Figure S1:

^{1}HNMR Structure **5**

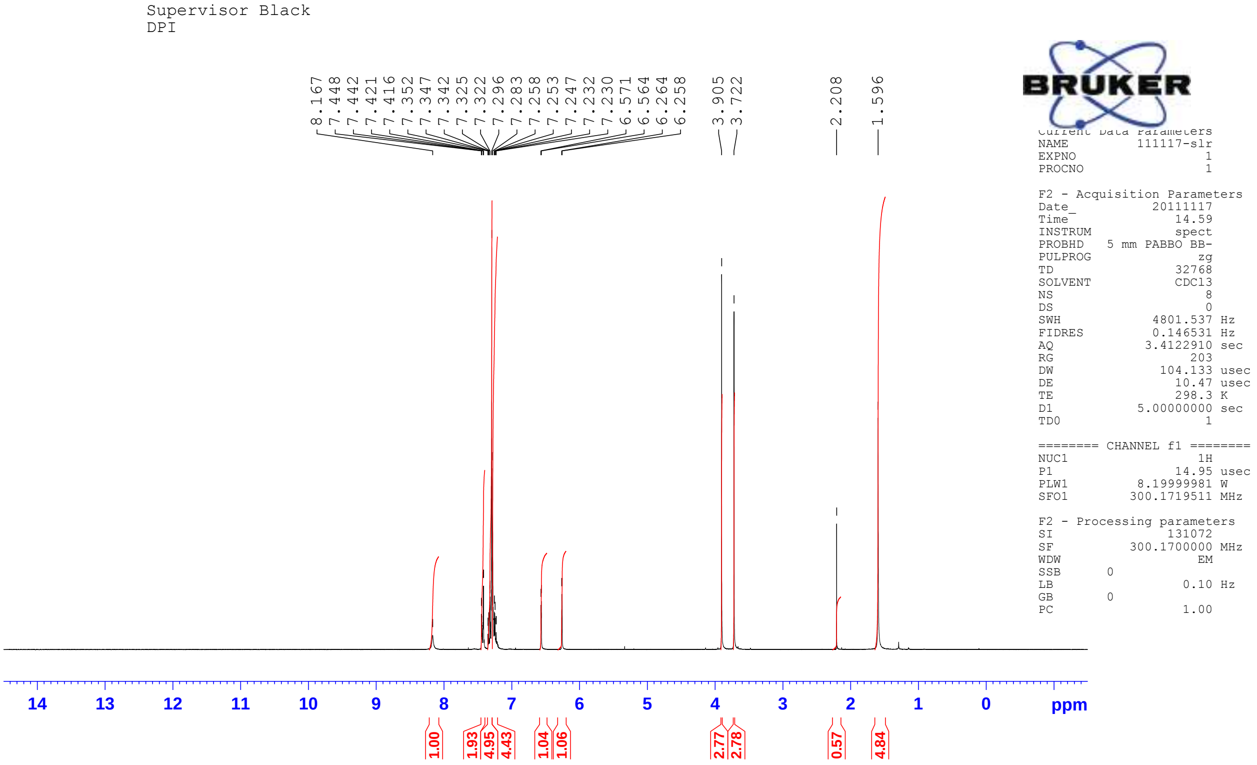

Figure S2:

^{1}HNMR Structure **7**

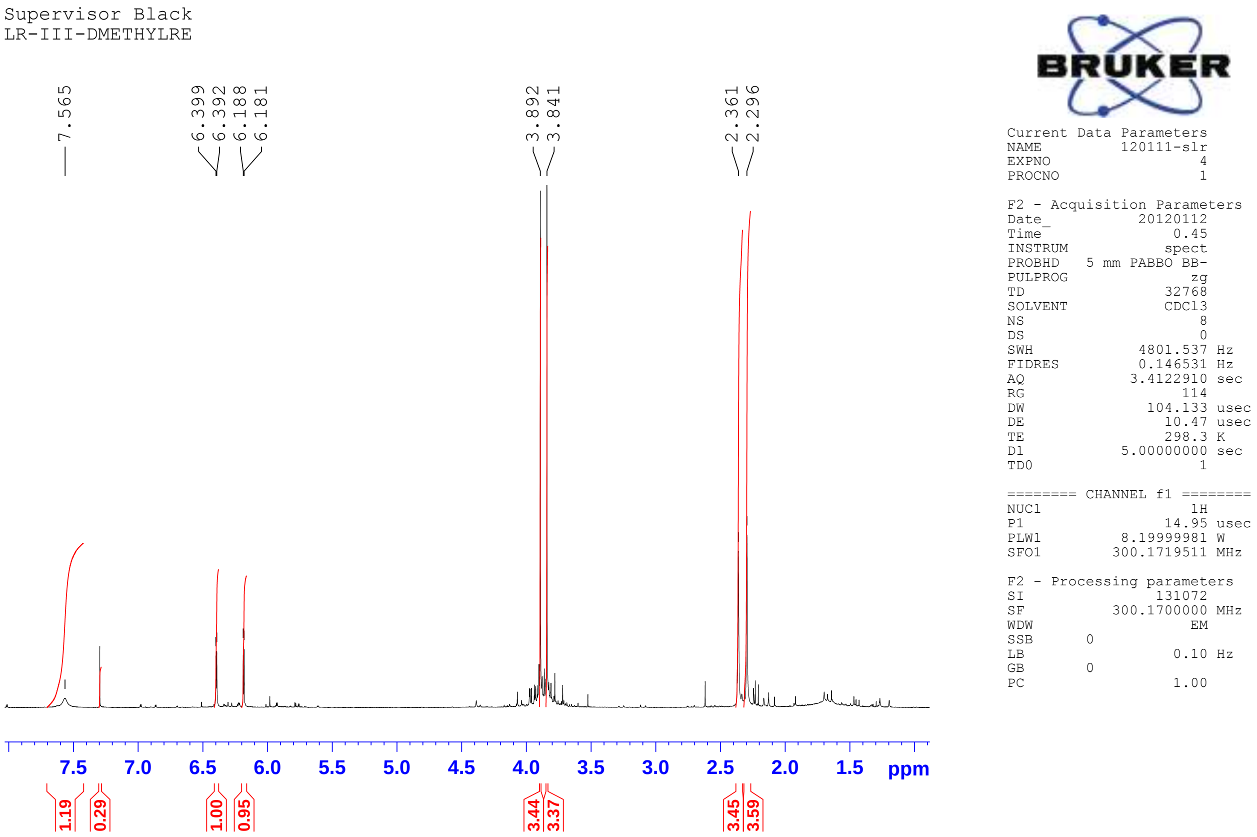

Figure S3:

^{13}CNMR Structure **7**

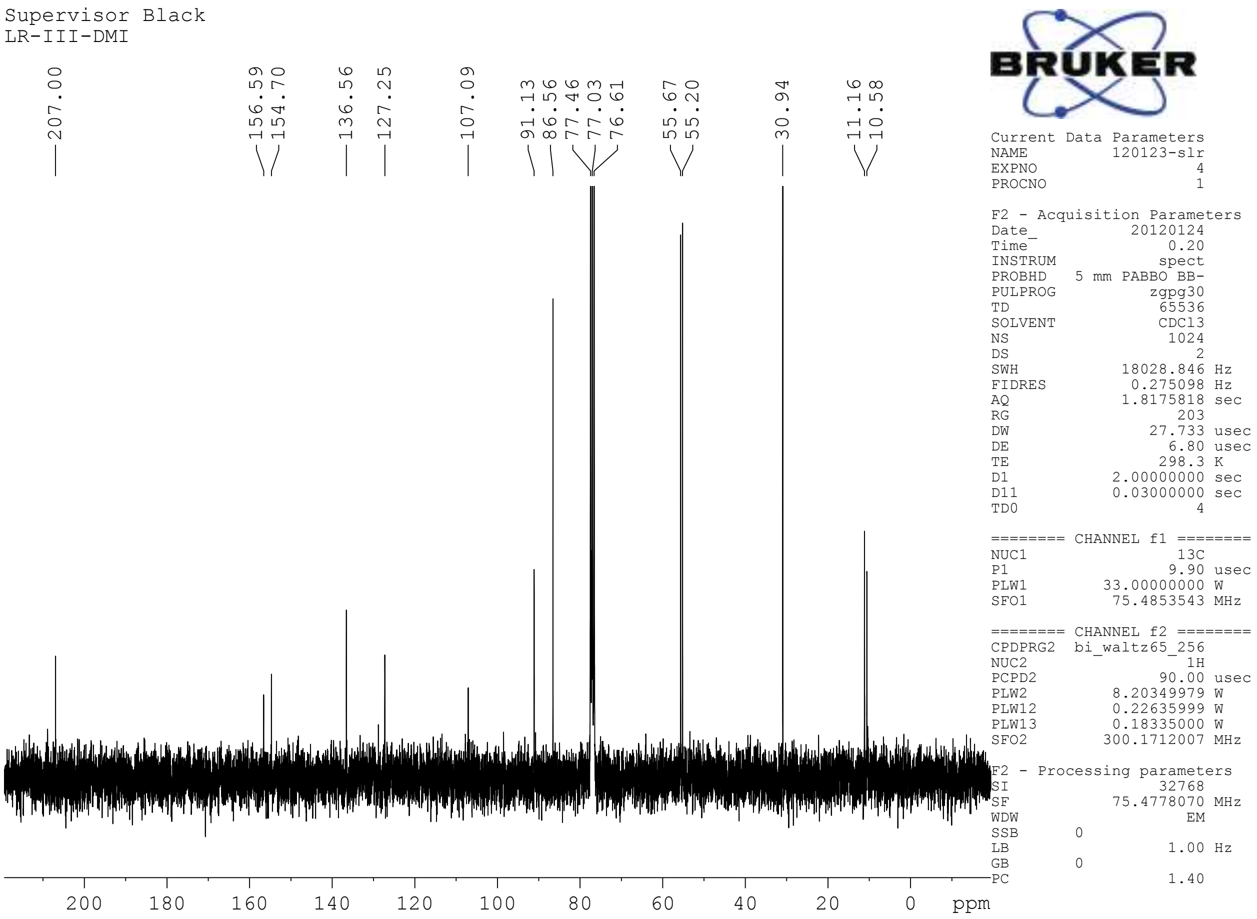

Figure S4:

^{1}HNMR Structure **8**

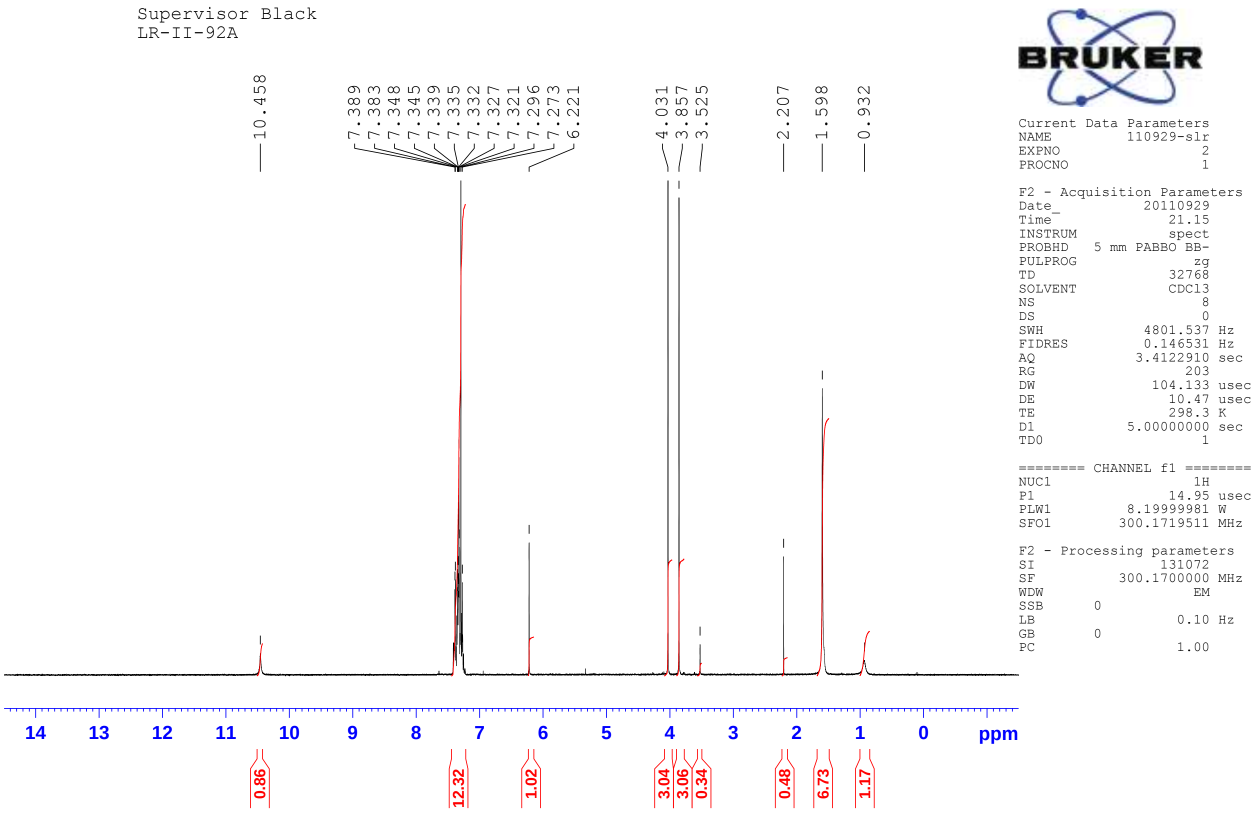

Figure S5:

^{1}HNMR Structure **9**

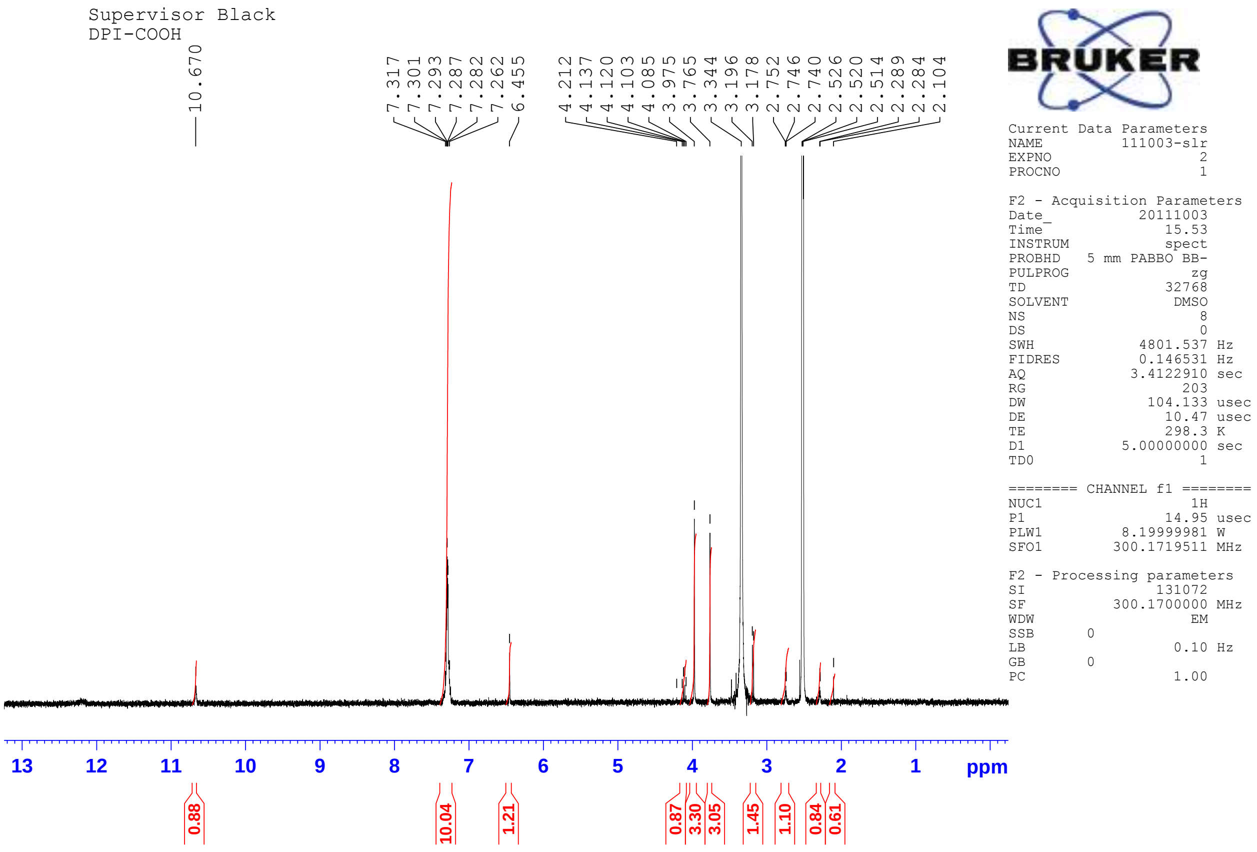

Figure S6:

^{13}CNMR Structure **10**

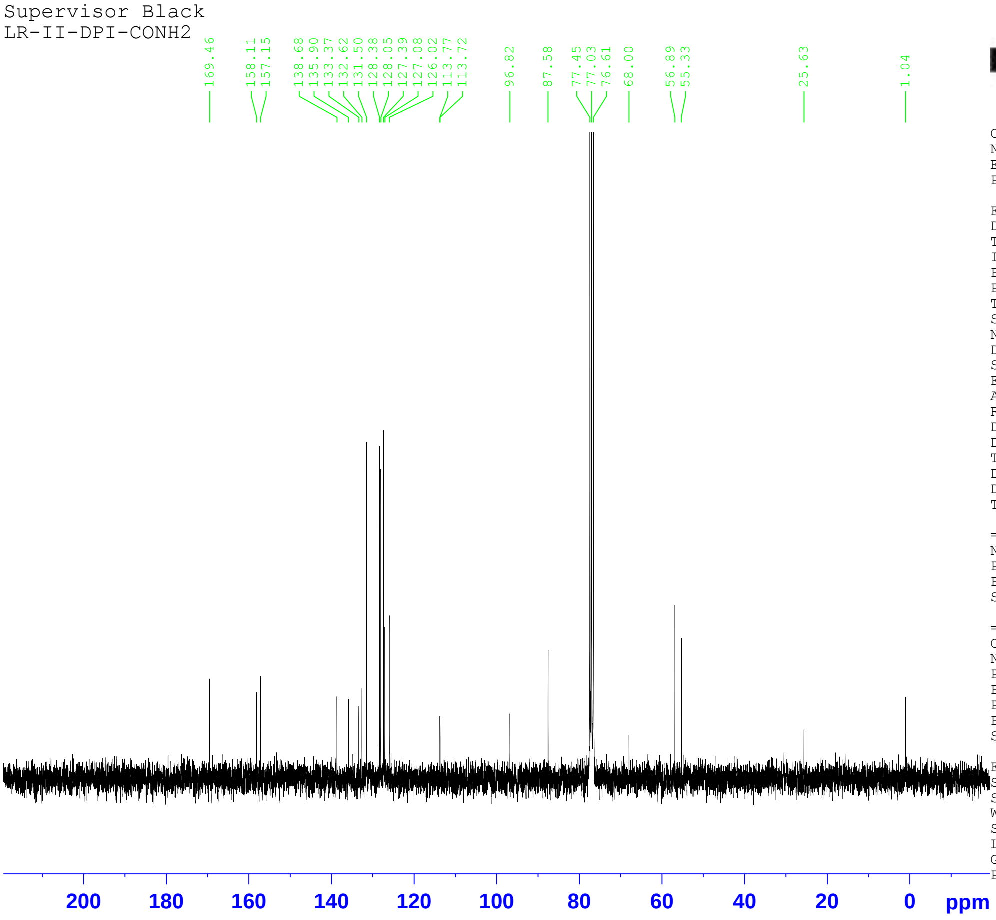

Current Data Parameters
NAME 111019-slr
EXPNO 1
PROCNO 1

F2 - Acquisition Parameters
Date_ 20111019
Time 17.39
INSTRUM spect
PROBHD 5 mm PABBO BB-
PULPROG zgpg30
TD 65536
SOLVENT CDCl3
NS 1024
DS 2
SWH 18028.846 Hz
FIDRES 0.275098 Hz
AQ 1.8175818 sec
RG 203
DW 27.733 usec
DE 6.80 usec
TE 298.3 K
D1 2.00000000 sec
D11 0.03000000 sec
TD0 4

======== CHANNEL f1 ========
NUC1 13C
P1 9.90 usec
PLW1 33.00000000 W
SFO1 75.4853543 MHz

======== CHANNEL f2 ========
CPDPRG2 bi_waltz65_256
NUC2 1H
PCPD2 90.00 usec
PLW2 8.20349979 W
PLW12 0.22635999 W
PLW13 0.18335000 W
SFO2 300.1712007 MHz

F2 - Processing parameters
SI 32768
SF 75.4778070 MHz
WDW EM
SSB 0
LB 1.00 Hz
GB 0
PC 1.40

Figure S7:

^{1}H NMR Structure **12** P#1

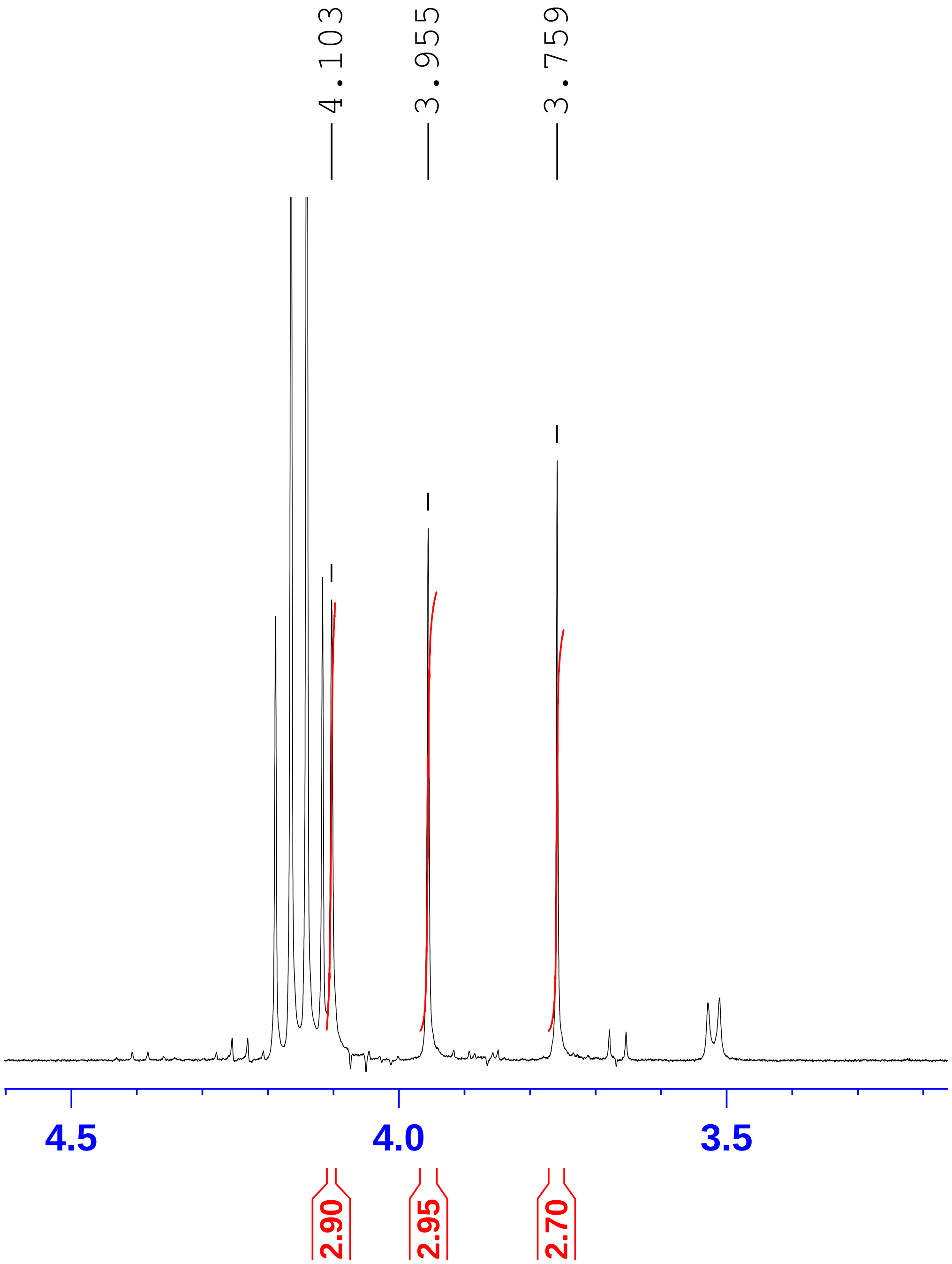

^{1}H NMR Structure **12** P#2

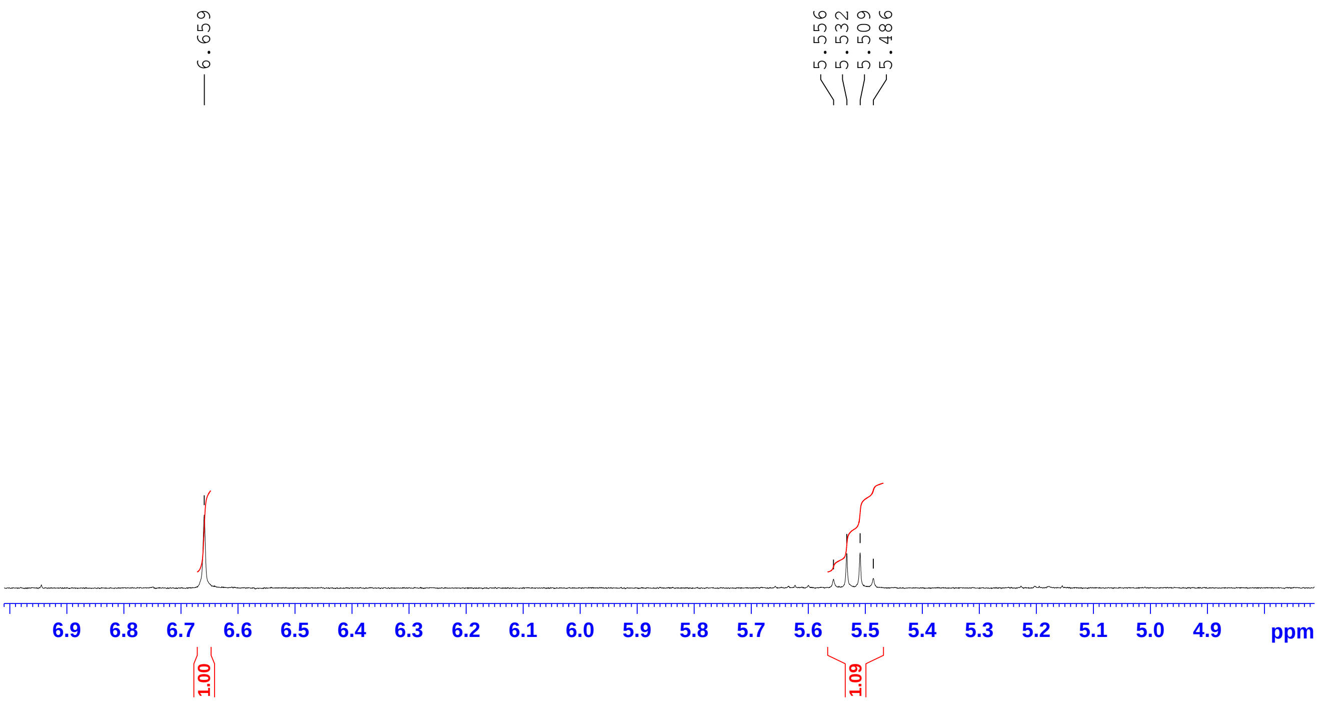

[1]NMR Structure **12** P#3+4

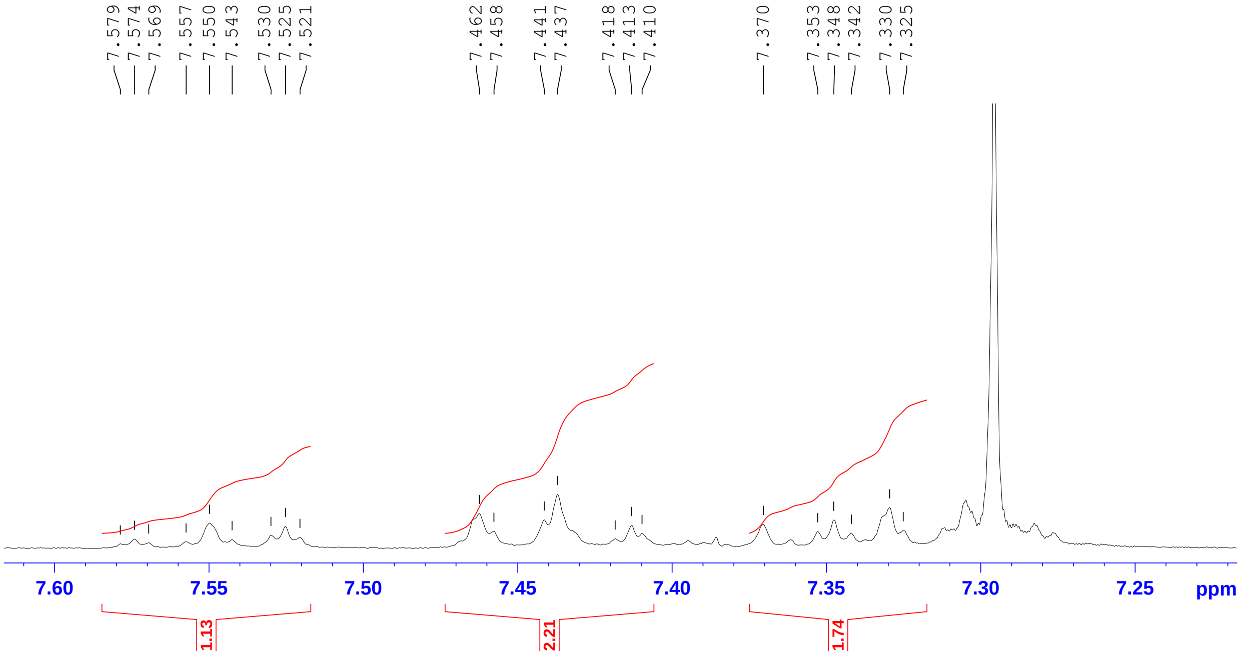

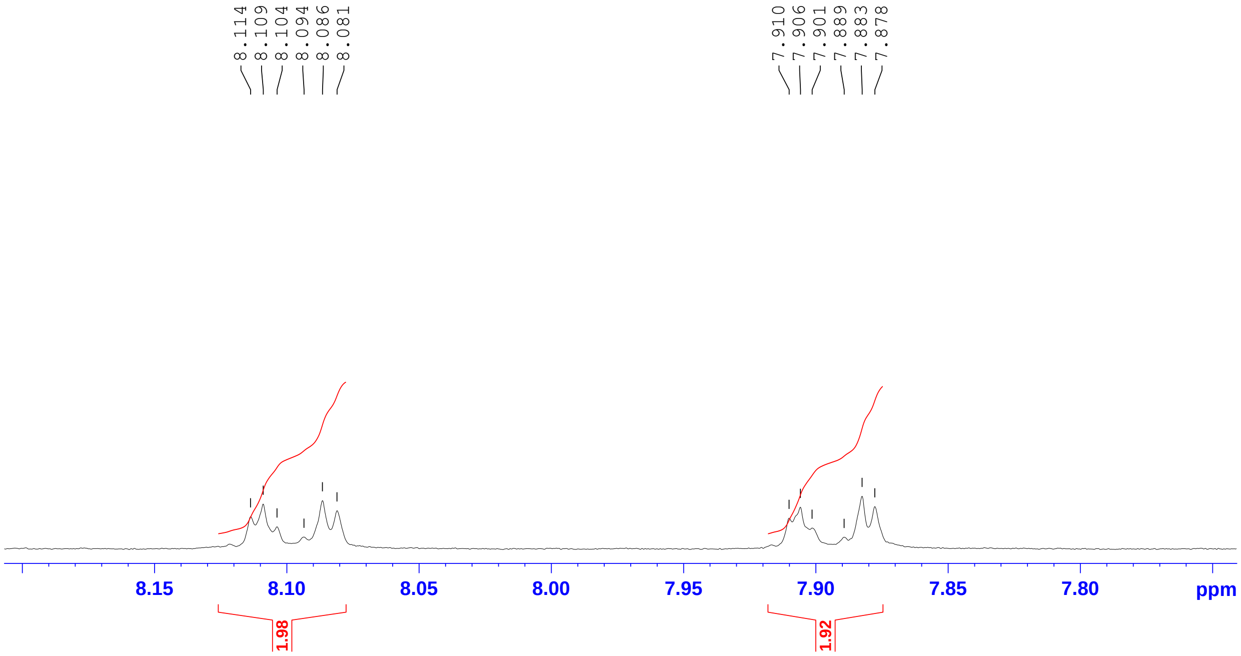

Figure S8:

^{13}CNMR Structure **12**

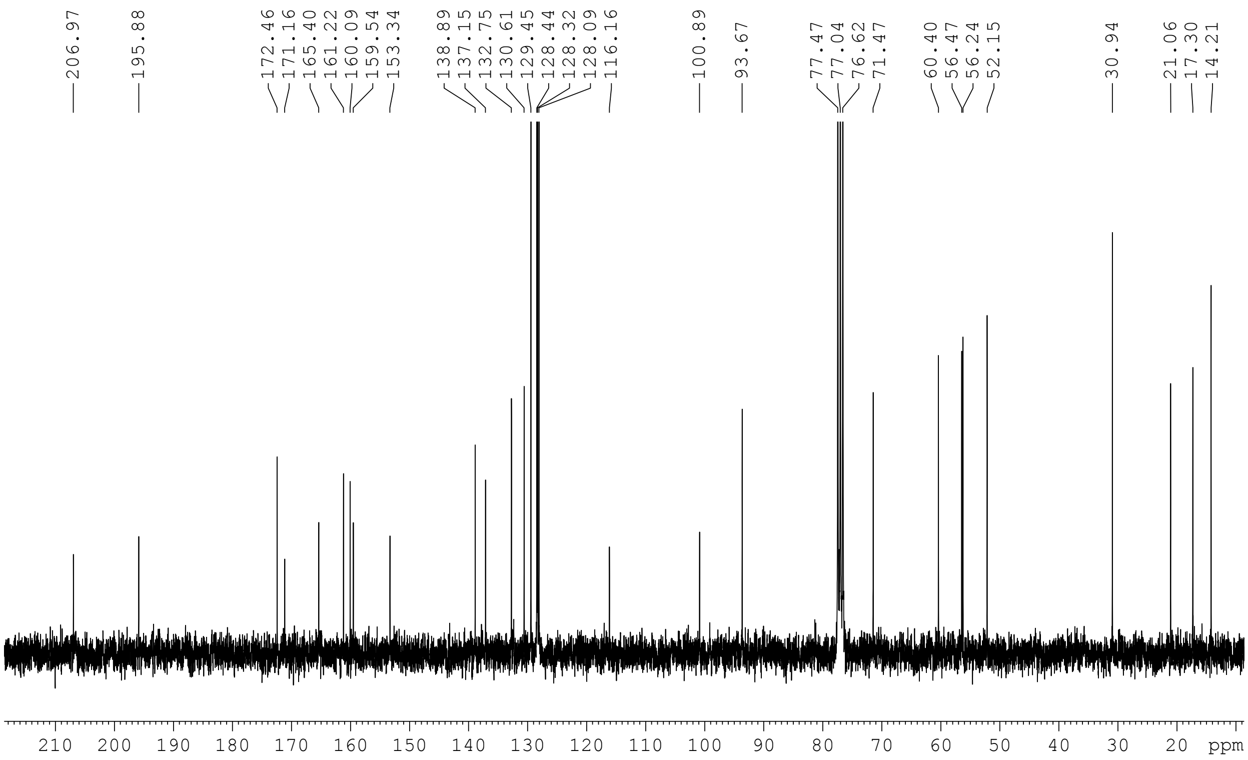

Figure S9: **MS Data from Orbitrap Strructure 12 P#1**

Tracking No: BMSF-2011-122
Samples Submitted By: Syeda Laila Rubab
Date Run: 26/10/11
Operator: Chowdhury Sarowar
Report Prepared By: Leanne Stephenson

Sample: LR-II.97A

Full Spectrum

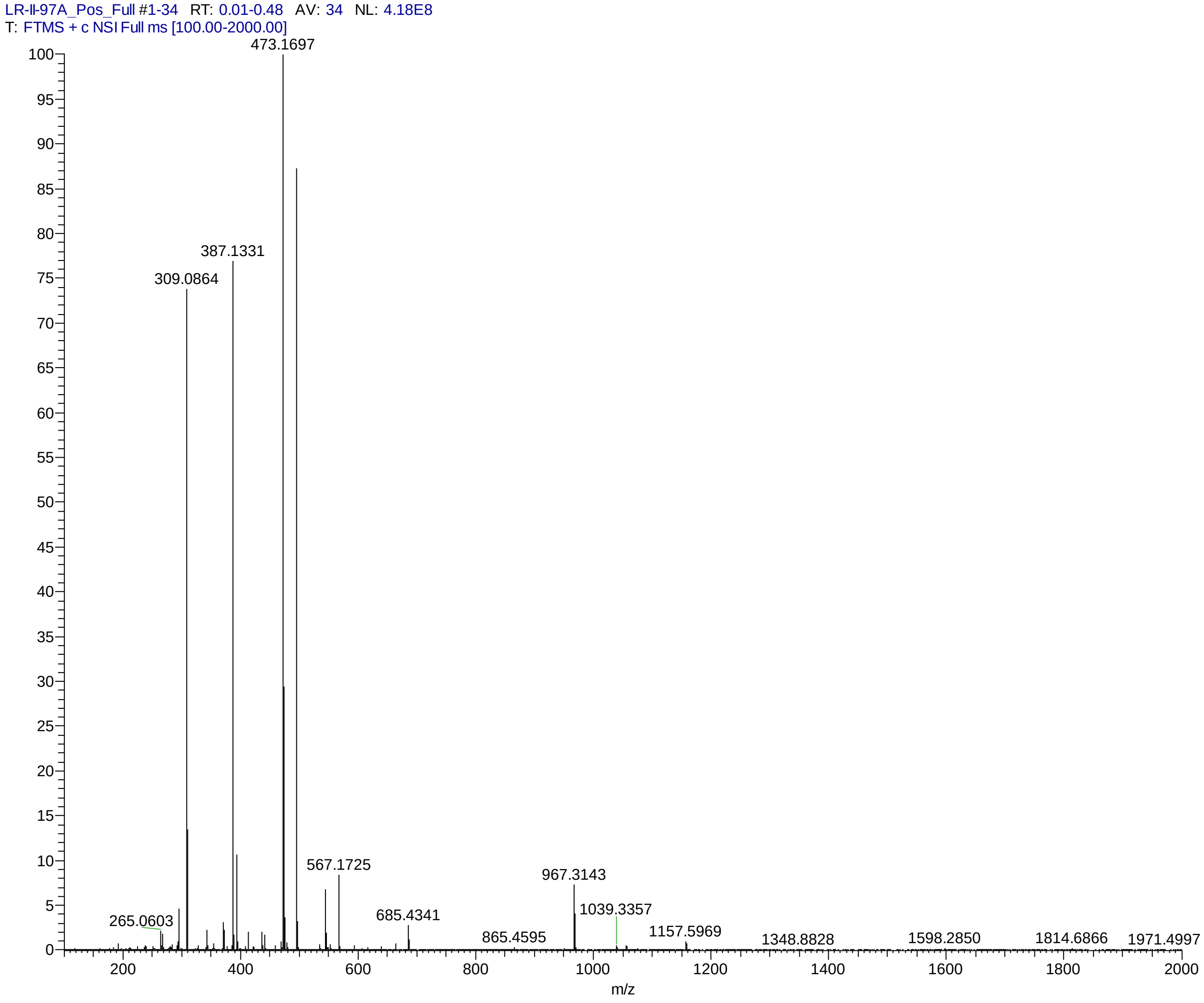

MS Data from Orbitrap Strructure 12 P#2

Zoomed Spectrum

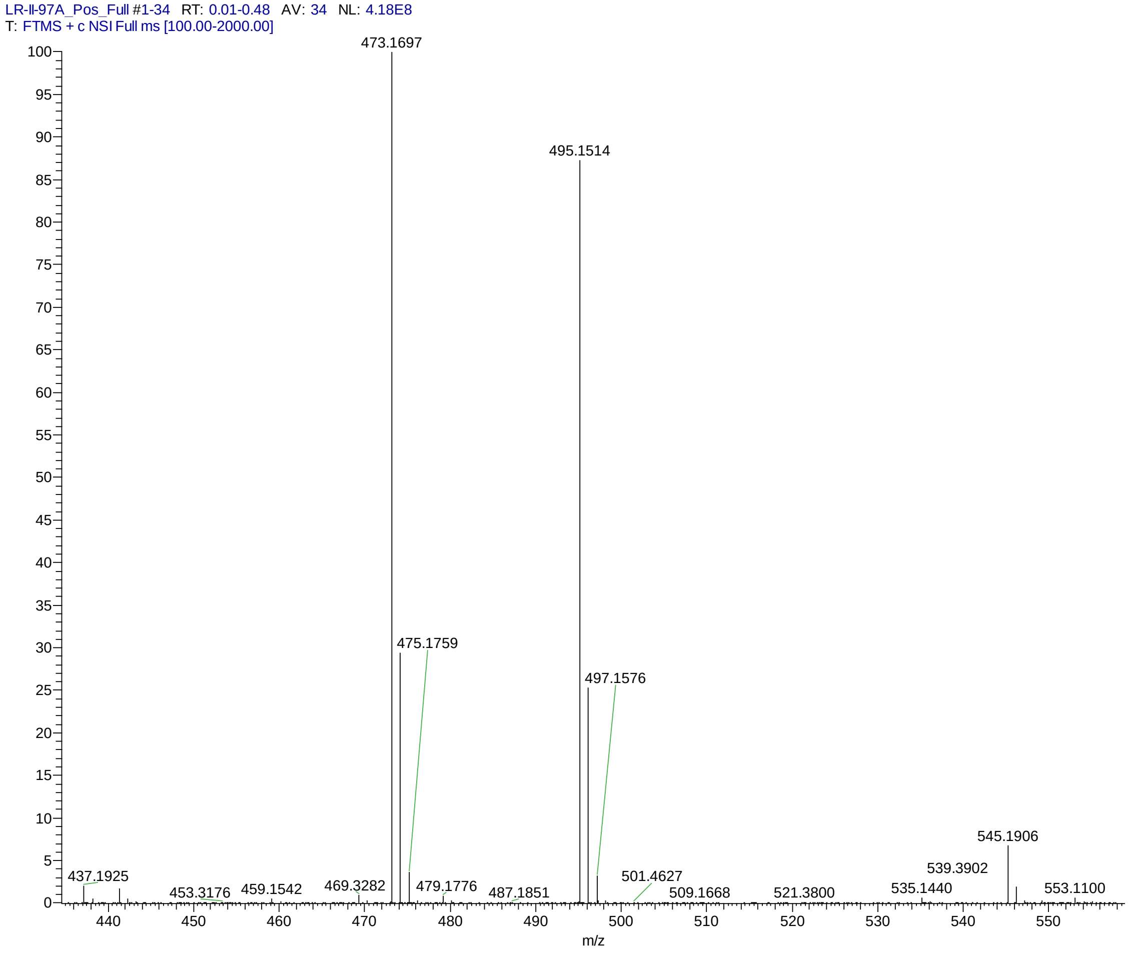

MS Data from Orbitrap Strructure 12 P#3

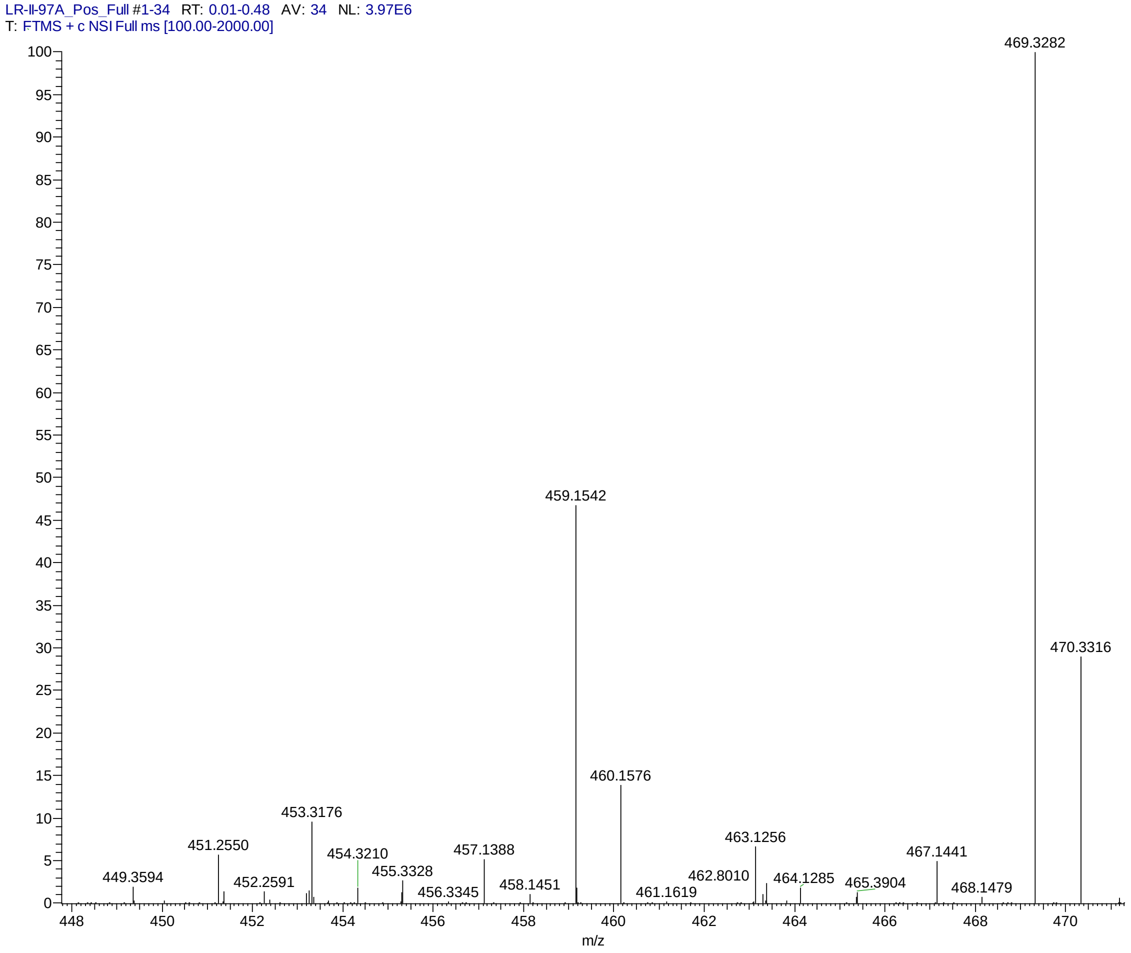

Figure S10: Remaining 2 D plots of **(a-d) 8**, **(e-h) 12**.
TA

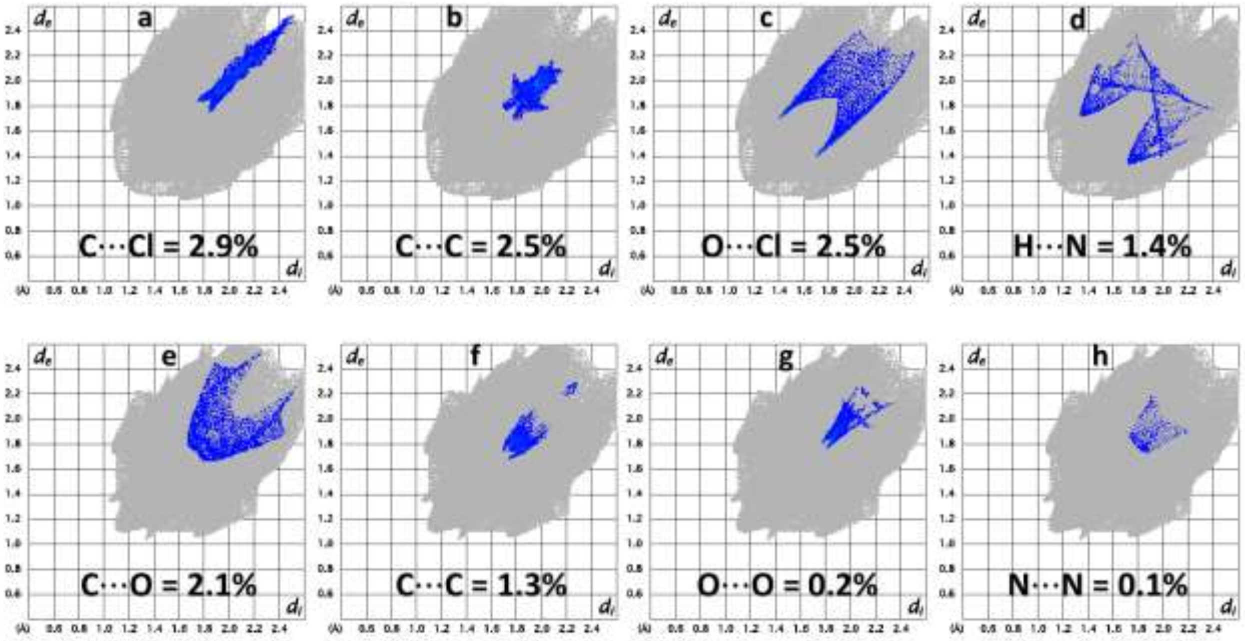

File S1:

checkCIF/PLATON report Structure 8

Structure factors have been supplied for datablock(s) LRIII92A

THIS REPORT IS FOR GUIDANCE ONLY. IF USED AS PART OF A REVIEW PROCEDURE FOR PUBLICATION, IT SHOULD NOT REPLACE THE EXPERTISE OF AN EXPERIENCED CRYSTALLOGRAPHIC REFEREE.

No syntax errors found. CIF dictionary Interpreting this report

Datablock: LRIII92A

```
Bond precision:   C-C = 0.0097 A          Wavelength=0.71073

Cell:               a=22.879(5)      b=6.0315(14)      c=16.084(4)
                    alpha=90         beta=90           gamma=90
Temperature:        296 K

                 Calculated                    Reported
Volume           2219.5(9)                     2219.5(9)
Space group      P c a 21                      P c a 21
Hall group       P 2c -2ac                     P 2c -2ac
Moiety formula   C24 H18 Cl3 N O3              C24 H18 Cl3 N O3
Sum formula      C24 H18 Cl3 N O3              C24 H18 Cl3 N O3
Mr               474.74                        474.74
Dx,g cm-3        1.421                         1.421
Z                4                             4
Mu (mm-1)        0.439                         0.439
F000             976.0                         976.0
F000'            978.15
h,k,lmax         27,7,19                       27,7,19
Nref             4135[ 2149]                   3218
Tmin,Tmax        0.910,0.936                   0.654,0.746
Tmin'            0.854

Correction method= # Reported T Limits: Tmin=0.654 Tmax=0.746
AbsCorr = MULTI-SCAN

Data completeness= 1.50/0.78       Theta(max)= 25.499

R(reflections)= 0.0450( 1588)                         wR2(reflections)=
                                                      0.0905( 3218)
S = 0.965                   Npar= 270
```

The following ALERTS were generated. Each ALERT has the format
test-name_ALERT_alert-type_alert-level.
Click on the hyperlinks for more details of the test.

Alert level C

```
PLAT026_ALERT_3_C Ratio Observed / Unique Reflections (too) Low ..        49% Check
PLAT242_ALERT_2_C Low    'MainMol' Ueq as Compared to Neighbors of        C22 Check
PLAT340_ALERT_3_C Low Bond Precision on  C-C Bonds ...............    0.00967 Ang.
PLAT911_ALERT_3_C Missing FCF Refl Between Thmin & STh/L=    0.600         10 Report
```

Alert level G

```
PLAT007_ALERT_5_G Number of Unrefined Donor-H Atoms .............          1 Report
PLAT431_ALERT_2_G Short Inter HL..A Contact  Cl3      ..O1       .       3.11 Ang.
                                                  x,-1+y,z  =      1_545 Check
PLAT912_ALERT_4_G Missing # of FCF Reflections Above STh/L=  0.600          2 Note
PLAT915_ALERT_3_G No Flack x Check Done: Low Friedel Pair Coverage         54 %
PLAT941_ALERT_3_G Average HKL Measurement Multiplicity ..........         4.4 Low
PLAT967_ALERT_5_G Note: Two-Theta Cutoff Value in Embedded .res ..       51.0 Degree
PLAT978_ALERT_2_G Number C-C Bonds with Positive Residual Density.          1 Info
```

0 **ALERT level A** = Most likely a serious problem - resolve or explain
0 **ALERT level B** = A potentially serious problem, consider carefully
4 **ALERT level C** = Check. Ensure it is not caused by an omission or oversight
7 **ALERT level G** = General information/check it is not something unexpected

0 ALERT type 1 CIF construction/syntax error, inconsistent or missing data
3 ALERT type 2 Indicator that the structure model may be wrong or deficient
5 ALERT type 3 Indicator that the structure quality may be low
1 ALERT type 4 Improvement, methodology, query or suggestion
2 ALERT type 5 Informative message, check

It is advisable to attempt to resolve as many as possible of the alerts in all categories. Often the minor alerts point to easily fixed oversights, errors and omissions in your CIF or refinement strategy, so attention to these fine details can be worthwhile. In order to resolve some of the more serious problems it may be necessary to carry out additional measurements or structure refinements. However, the purpose of your study may justify the reported deviations and the more serious of these should normally be commented upon in the discussion or experimental section of a paper or in the "special_details" fields of the CIF. checkCIF was carefully designed to identify outliers and unusual parameters, but every test has its limitations and alerts that are not important in a particular case may appear. Conversely, the absence of alerts does not guarantee there are no aspects of the results needing attention. It is up to the individual to critically assess their own results and, if necessary, seek expert advice.

Publication of your CIF in IUCr journals

A basic structural check has been run on your CIF. These basic checks will be run on all CIFs submitted for publication in IUCr journals (*Acta Crystallographica*, *Journal of Applied Crystallography*, *Journal of Synchrotron Radiation*); however, if you intend to submit to *Acta Crystallographica Section C* or *E* or *IUCrData*, you should make sure that full publication checks are run on the final version of your CIF prior to submission.

Publication of your CIF in other journals

Please refer to the *Notes for Authors* of the relevant journal for any special instructions relating to CIF submission.

PLATON version of 28/11/2022; check.def file version of 28/11/2022

Datablock LRIII92A - ellipsoid plot

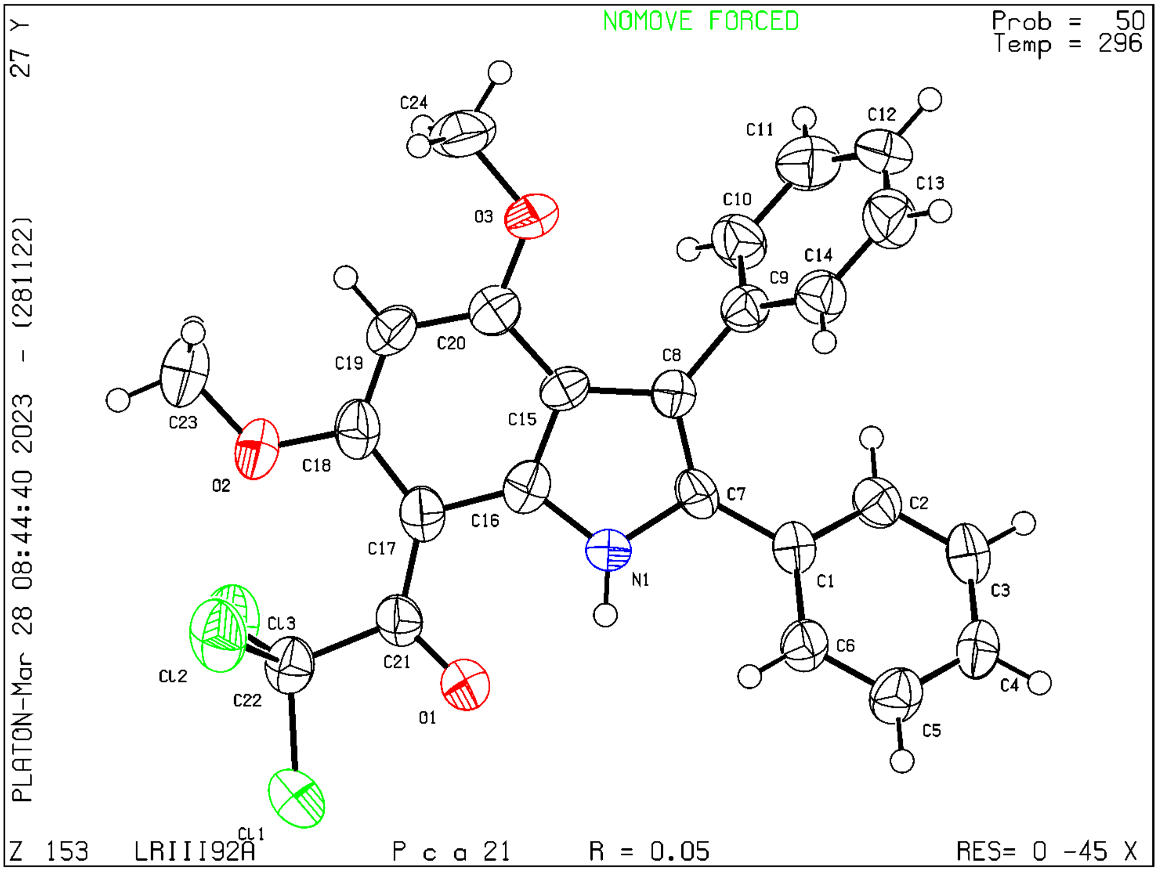

File S2:

checkCIF/PLATON report Structure 12

Structure factors have been supplied for datablock(s) LR_III_9730_0m

THIS REPORT IS FOR GUIDANCE ONLY. IF USED AS PART OF A REVIEW PROCEDURE FOR PUBLICATION, IT SHOULD NOT REPLACE THE EXPERTISE OF AN EXPERIENCED CRYSTALLOGRAPHIC REFEREE.

No syntax errors found. CIF dictionary Interpreting this report

Datablock: LR_III_9730_0m

```
Bond precision:   C-C = 0.0047 A          Wavelength=0.71073

Cell:             a=14.3363(16)     b=12.0884(13)       c=14.8394(17)
                  alpha=90          beta=109.053(6)     gamma=90
Temperature:      296 K

                  Calculated                      Reported
Volume            2430.8(5)                       2430.8(5)
Space group       P 21/n                          P 21/n
Hall group        -P 2yn                          -P 2yn
Moiety formula    C27 H24 N2 O6                   C27 H24 N2 O6
Sum formula       C27 H24 N2 O6                   C27 H24 N2 O6
Mr                472.48                          472.48
Dx,g cm-3         1.291                           1.291
Z                 4                               4
Mu (mm-1)         0.092                           0.092
F000              992.0                           992.0
F000'             992.51
h,k,lmax          17,14,18                        17,14,18
Nref              4780                            4764
Tmin,Tmax         0.968,0.978                     0.903,0.950
Tmin'             0.968

Correction method= # Reported T Limits: Tmin=0.903 Tmax=0.950
AbsCorr = MULTI-SCAN

Data completeness= 0.997           Theta(max)= 25.999

                                                    wR2(reflections)=
R(reflections)= 0.0580( 2022)                       0.1441( 4764)

S = 0.985                    Npar= 341
```

The following ALERTS were generated. Each ALERT has the format
test-name_ALERT_alert-type_alert-level.
Click on the hyperlinks for more details of the test.

Alert level C

```
PLAT026_ALERT_3_C Ratio Observed / Unique Reflections (too) Low ..        42% Check
PLAT213_ALERT_2_C Atom O6B             has ADP max/min Ratio .....        3.2 prolat
PLAT220_ALERT_2_C NonSolvent   Resd 1  C   Ueq(max)/Ueq(min) Range        5.1 Ratio
PLAT222_ALERT_3_C NonSolvent Resd 1  H   Uiso(max)/Uiso(min) Range        5.1 Ratio
PLAT242_ALERT_2_C Low    'MainMol' Ueq as Compared to Neighbors of        C26 Check
PLAT340_ALERT_3_C Low Bond Precision on  C-C Bonds ..............     0.00467 Ang.
PLAT906_ALERT_3_C Large K Value in the Analysis of Variance ......     16.874 Check
PLAT906_ALERT_3_C Large K Value in the Analysis of Variance ......      2.720 Check
PLAT911_ALERT_3_C Missing FCF Refl Between Thmin & STh/L=    0.600          8 Report
```

Alert level G

```
PLAT002_ALERT_2_G Number of Distance or Angle Restraints on AtSite          4 Note
PLAT003_ALERT_2_G Number of Uiso or Uij Restrained non-H Atoms ...          4 Report
PLAT172_ALERT_4_G The CIF-Embedded .res File Contains DFIX Records          1 Report
PLAT186_ALERT_4_G The CIF-Embedded .res File Contains ISOR Records          2 Report
PLAT301_ALERT_3_G Main Residue  Disorder ..............(Resd  1  )         6% Note
PLAT721_ALERT_1_G Bond    Calc     0.97000, Rep     0.96000 Dev...       0.01 Ang.
              C27B     -H27D            1_555   1_555 ........     #   65 Check
PLAT793_ALERT_4_G Model has Chirality at C24         (Centro SPGR)          R Verify
PLAT860_ALERT_3_G Number of Least-Squares Restraints ............          26 Note
PLAT912_ALERT_4_G Missing # of FCF Reflections Above STh/L=  0.600          8 Note
PLAT941_ALERT_3_G Average HKL Measurement Multiplicity ..........         4.0 Low
PLAT967_ALERT_5_G Note: Two-Theta Cutoff Value in Embedded .res ..       52.0 Degree
PLAT978_ALERT_2_G Number C-C Bonds with Positive Residual Density.          1 Info
```

0 **ALERT level A** = Most likely a serious problem - resolve or explain
0 **ALERT level B** = A potentially serious problem, consider carefully
9 **ALERT level C** = Check. Ensure it is not caused by an omission or oversight
12 **ALERT level G** = General information/check it is not something unexpected

1 ALERT type 1 CIF construction/syntax error, inconsistent or missing data
6 ALERT type 2 Indicator that the structure model may be wrong or deficient
9 ALERT type 3 Indicator that the structure quality may be low
4 ALERT type 4 Improvement, methodology, query or suggestion
1 ALERT type 5 Informative message, check

It is advisable to attempt to resolve as many as possible of the alerts in all categories. Often the minor alerts point to easily fixed oversights, errors and omissions in your CIF or refinement strategy, so attention to these fine details can be worthwhile. In order to resolve some of the more serious problems it may be necessary to carry out additional measurements or structure refinements. However, the purpose of your study may justify the reported deviations and the more serious of these should normally be commented upon in the discussion or experimental section of a paper or in the "special_details" fields of the CIF. checkCIF was carefully designed to identify outliers and unusual parameters, but every test has its limitations and alerts that are not important in a particular case may appear. Conversely, the absence of alerts does not guarantee there are no aspects of the results needing attention. It is up to the individual to critically assess their own results and, if necessary, seek expert advice.

Publication of your CIF in IUCr journals

A basic structural check has been run on your CIF. These basic checks will be run on all CIFs submitted for publication in IUCr journals (*Acta Crystallographica*, *Journal of Applied Crystallography*, *Journal of Synchrotron Radiation*); however, if you intend to submit to *Acta Crystallographica Section C* or *E* or *IUCrData*, you should make sure that full publication checks are run on the final version of your CIF prior to submission.

Publication of your CIF in other journals

Please refer to the *Notes for Authors* of the relevant journal for any special instructions relating to CIF submission.

PLATON version of 28/11/2022; check.def file version of 28/11/2022

Datablock LR_III_9730_0m - ellipsoid plot

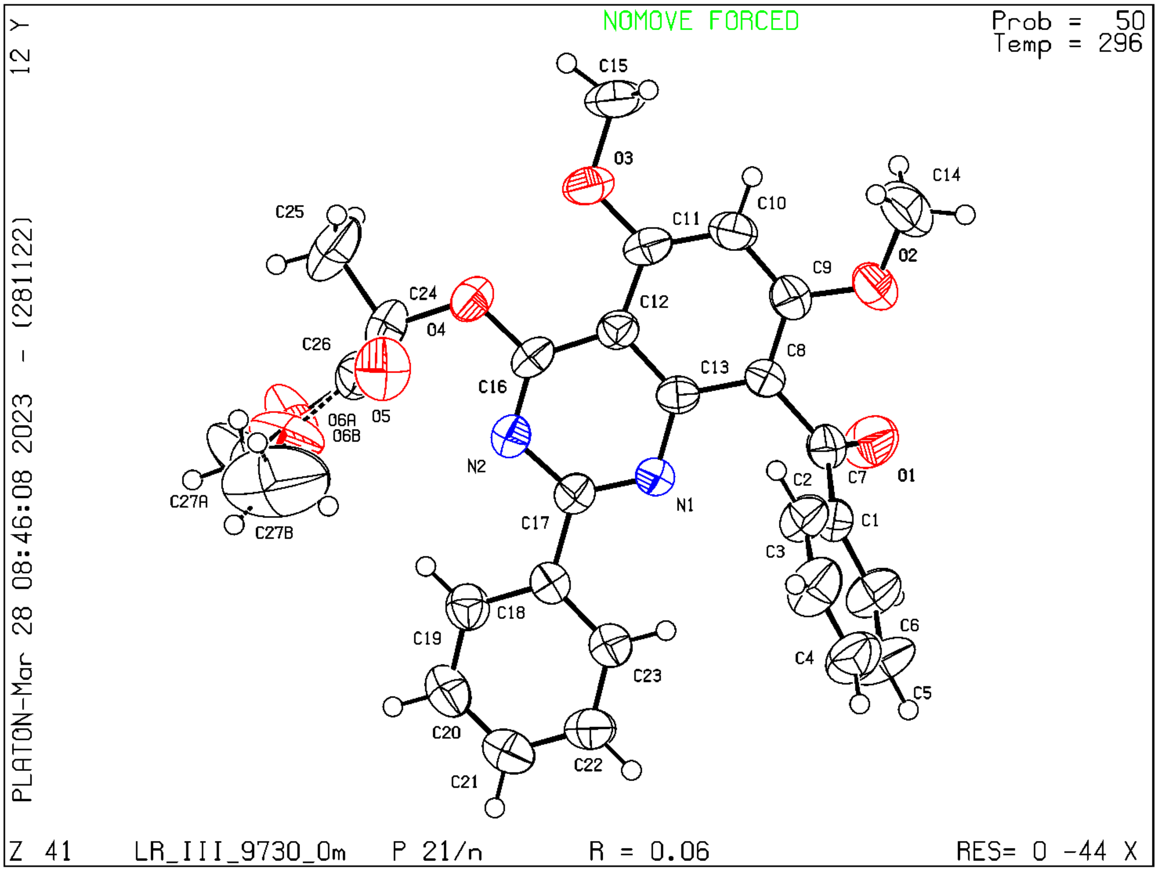

Supplement: Supplementary file 1 [file molecules-28-05024-s001.zip › molecules-2423186-supplementary.pdf]
